# Sexual dimorphism in peri-articular tissue anatomy – More keys to understanding sex-differences in osteoarthritis?

**DOI:** 10.1016/j.ocarto.2024.100485

**Published:** 2024-05-11

**Authors:** Felix Eckstein, Reinhard Putz, Wolfgang Wirth

**Affiliations:** a Research Program for Musculoskeletal Imaging, Center for Anatomy and Cell Biology, Paracelsus Medical University, Salzburg, Austria; b Ludwig Boltzmann Institute for Arthritis and Rehabilitation (LBIAR), Paracelsus Medical University, Salzburg, Austria; c Chondrometrics GmbH, Ainring, Germany; d Anatomische Anstalt, Ludwig Maximilians Universität München, Munich, Germany

**Keywords:** Peri-articular tissue, Sex, Joint, Knee, Osteoarthritis

## Abstract

**Objective:**

Osteoarthritis prevalence differs between women and men; whether this is the result of differences in pre-morbid articular or peri-articular anatomical morphotypes remains enigmatic. Albeit sex within humans cannot be reduced to female/male, this review focusses to the sexual dimorphism of peri-articular tissues, given lack of literature on non-binary subjects.

**Methods:**

Based on a Pubmed search and input from experts, we selected relevant articles based on the authors’ judgement of relevance, interest, and quality; no “hard” bibliometric measures were used to evaluate the quality or importance of the work. Emphasis was on clinical studies, with most (imaging) data being available for the knee and thigh.

**Results:**

The literature on sexual dimorphism of peri-articular tissues is reviewed: 1) bone size/shape, 2) subchondral/subarticular bone, 3) synovial membrane and infra-patellar fad-pad (IPFP), 4) muscle/adipose tissue, and 5) peri-articular tissue response to treatment.

**Conclusions:**

Relevant sex-specific differences exist for 3D bone shape and IPFP size, even after normalization to body weight. Presence of effusion- and Hoffa-synovitis is associated with greater risk of incident knee osteoarthritis in overweight women, but not in men. When normalized to bone size, men exhibit greater muscle, and women greater adipose tissue measures relative to the opposite sex. Reduced thigh muscle specific strength is associated with incident knee osteoarthritis and knee replacement in women, but not in men. These observations may explain why women with muscle strength deficits have a poorer prognosis than men with similar deficits. A “one size/sex fits all” approach must be urgently abandoned in osteoarthritis research.

## 1 Introduction

Sex-differences in morphological, physiological, molecular, pathological, or behavioral traits are key in clinical medicine, to reliably prevent, diagnose, and treat disease, and make accurate prognoses. In osteoarthritis (OA), evaluation and treatment of joint pain and structure requires particular consideration of sex, since being female is an important risk factor [1], albeit female/male prevalence/incidence ratios vary substantially between anatomical locations [2].

In a parallel review, we have delineated sex-differences of articular tissues, including radiographic joint space width, meniscus, ligaments, and articular cartilage [3]. Peri-articular tissues, however, were not included, and his review therefore focuses on the sexual dimorphisms of these anatomical structures. We confered that the “one size/sex fits all” approach should be left behind in OA research and scientific reporting, and that sexual diversity (biologically and socially) is to be recognized in research and clinical practice [4]. As we here report on biological (and not sociological) features, we employ the term “sex” rather than “gender”. We will differentiate between “observed” and “genuine” sex-differences, the former not accounting for differences in body constitution, whereas the latter being independent of such confounders.

Reflections on sex-differences in morphology must start with considerations of heterogeneity within populations. Humans display relatively homogeneous phenotypes compared with other species [5], whereas dogs, for instance, exhibit outstanding heterogeneity in size, shape, and (joint) pathology, partly due to specific breeding. The German shepherd exemplifies how polygenetic disposition can cause hip dysplasia, excessive joint laxity, and subluxation, leading to extraordinary biomechanical stress, hip OA and joint failure, whereas other dog breeds do not share this fate.

The concept of sexual dimorphism represents a first step in creating “types” within a heterogeneous population, cognizant that individuals should not be regarded or treated the same (personalized medicine), but that current medicine is unable to treat each individual differently. Sex differentiation should, however, not exclude non-binary individuals, on whom scientific literature in OA is sparse to absent.

One of the best-studied sexual dimorphism is body size [6], ranging from extremely female-biased (female>males, e.g. spiders [7]) to male-biased (e.g. elephant seals [8]). Plumage coloration is another example, male birds being more colorful than their female counterparts [9]. Humans, for instance, display greater sexual dimorphism of the pelvic inlet than gibbon and tamarin monkeys, despite demonstrating a lower relative newborn body mass than these primates [10]. Human sex-differences are also noted in the peripheral skeleton and help identifying female/male footprints in wild-ranging animals. Even in species with minimal sexual dimorphism non-detectable to the naked eye, such as the giant panda, male/female footprints can be identified when sophisticated statistical identification methods are employed [11].

Currently it is unclear to what extent sex-differences in OA prevalence and severity originate from sex-differences of patho-physiological mechanisms or sexual dimorphisms in joint anatomy, potentially exposing women to greater vulnerability and risk of tissue damage. Expanding on our parallel review [3], we here focus on peri-articular tissues, mainly around the knee, relying predominantly on the use of modern imaging technology. Given recommendations on the need to also study healthy children, adolescents and adults prior to OA incidence [12], we will highlight findings in pre-morbid subjects, where available.

## 2 Methods

We performed a Pubmed search, using a variety of search terms, including sex, women/men, female/male, dimorphism, limbs, joint, peri-articular tissue, radiography, CT, magnetic resonance imaging, knee, bone, subchondral, subarticular, trabecular, synovial membrane, synovitis, effusion, IPFP, muscle, fat, adipose tissue, intervention. The search was not limited to a certain publication period. We further contacted experts in the field, asking for assistance in identifying and interpreting relevant literature (acknowledgement). References were included/excluded based on the authors’ judgement of their relevance and interest to the topic, their scientific quality (e.g. properness of the analytic methods used, the sample size, statistical testing procedures etc.), and their originality. No “hard” objective, bibliometric measure was used to evaluate the importance or quality of the work. Emphasis was on clinical studies in humans. We acknowledge existence of other topics relevant to understanding sex-differences of OA that were not included.

## 3 Results

i. Bone Size and Shape

The term “Osteo”-Arthritis stresses the role of bone in the disease, although not directly part of the joint articulation. Bones compose the human skeleton, transferring mechanical loads from one joint to the next. They provide the foundation of the cartilaginous joint surface and become more directly involved when mechanically overloaded (osteophytes), particularly when cartilage dissolves.

Sexual dimorphism may affect bone size and shape, the relative position of bones (parts) to each other (e.g. valgus/varus), or trabecular/cortical (micro-) structure. Regarding bone size, a different length ratio between the 2nd (2D) vs. 4th digit (4D) was described >100 years ago as sex-dependent trait in tetrapods, including birds, reptiles, amphibians, and mammals [13]. This sexual dimorphism in “relative” bone size dates back not less than 400 million years [14]. In humans, the 2D:4D ratio is lower in men; this is similar for some species (e.g. lizards, rodents, chimpanzees), but it is lower in women in others (e.g. frogs, birds, rhesus monkeys, guinea baboons) [15,16]. This dimorphism appears to be associated with fetal exposure to maternal sex steroids and patterns of testosterone/estrogen receptor density in most species [17,18].

Men display larger bones and bone-cartilage-interface areas than women [19], the latter sex-difference apparently being “genuine” [3]. In adolescents, the annual gain in the femorotibial interface area was 0.4/0.7% in boys/girls (no statistical difference) [19] with similar findings in the patella [20]. In radiographically normal knees from participants of the Osteoarthritis Initiative (OAI) [21,22], body height was the strongest demographic determinant of variance of medial femorotibial bone-cartilage-interface area in each sex. But even together with age and weight, it only explained 55% of the variability in women, and 30% in men [23]. The size of the bone-cartilage-interface was observed to increase in knee OA (RKOA), stronger than that in healthy controls; sex, BMI, and knee alignment had, however, only small effects on the rates of change [24].

No sex-differences in MRI-signs of bone pathology were observed in participants without RKOA in a population-based study (Framingham), with osteophytes in 72% women and in 77% men (p ​= ​0.12), and bone attrition (flattening or depression of the bone-cartilage-interface) in 32% vs. 33% (p ​= ​0.72) [25]. This was confirmed when stricter reading definitions were applied [25], and in a meta-analysis of >3000 uninjured knees [26].

**Fig. 1:**
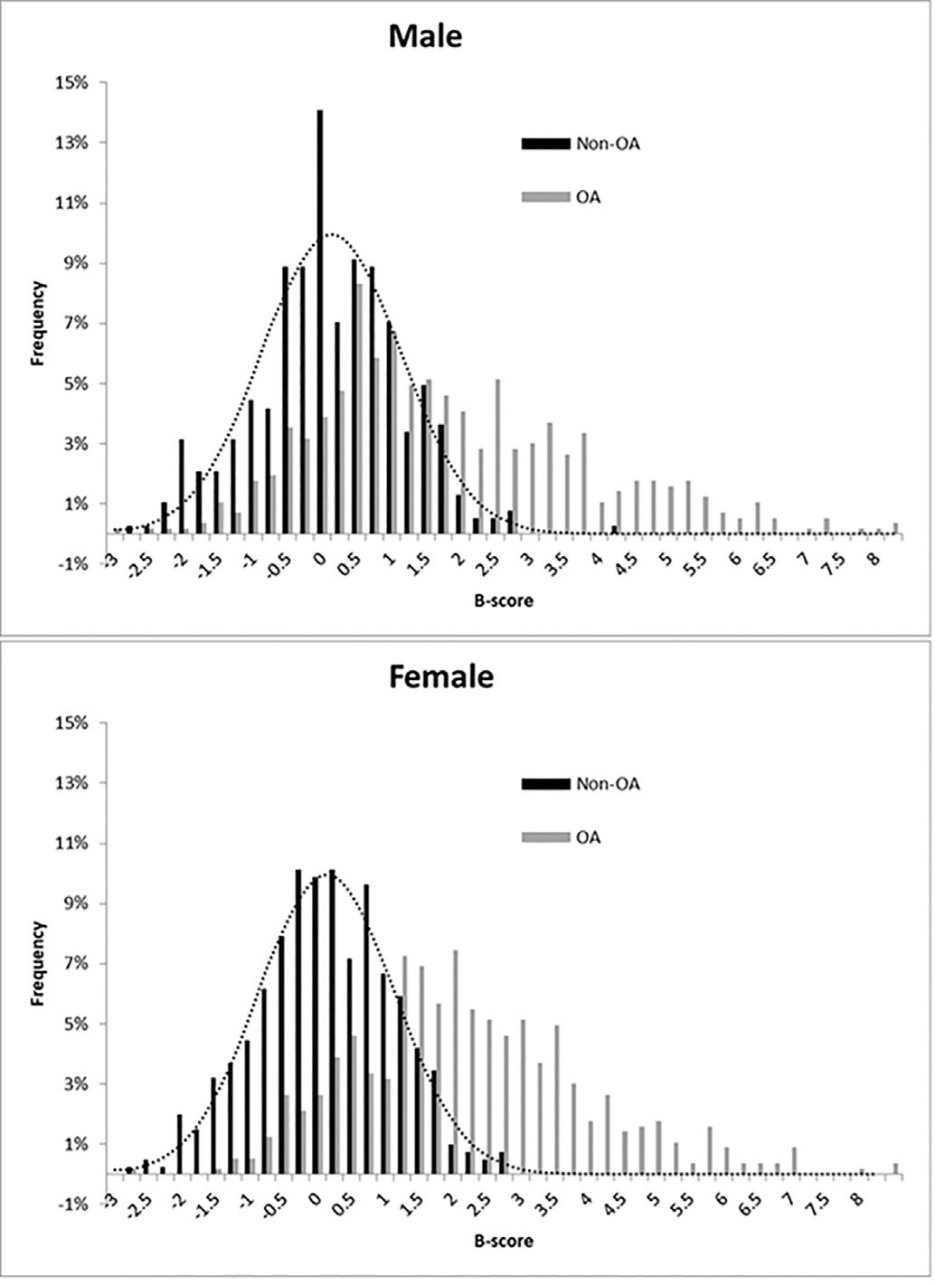
Distribution of bone shape of Non-OA and OA patients following correction of male-female B-score means: A normal distribution with a mean value of 0 and a standard deviation of 1 is shown by the dotted line in each histogram. Both women and men from the Non-OA group are normally distributed along the OA vector, centered at zero after correction for sex. Graphs kindly provided by Alan Brett, Imorphics, Manchester, UK.

Femoropatellar 3D MRI bone shape (determined using active appearance models) significantly differed between women and men (classification accuracy 91%; Fig. 1), but did not predict join pain status [27]. The B-score represents an encapsulating measure of 3D bone shape [24], the origin (0) being defined separately for men/women by sex-specific mean shapes of non-OA groups. The OA vector denotes the itinerary of bone shape change with OA progression and is constructed for women and men together, as shape changes were observed similar between sexes [24] (Fig. 1). Women were reported to display greater odds of incident RKOA then men, with an inconsistent mediation effect for radiographic bone shape, potentially preventing even greater risk in women [28]. This analysis also found the angulation of the femur and tibia involved (valgus/varus) [28].

Knee alignment in healthy subjects depends on sex and race: Values in the Framingham cohort were slight valgus in women (1.84°), and more valgus in men (3.44°) [29], whereas in the Bejing cohort, sex-differences in alignment were smaller (0.94°), men/women being more valgus than Caucasians [29]. Femorotibial alignment from short-limb knee radiographs was found skewed towards varus compared with long-limb measures, the difference being stronger in women than men (4.8° vs. 3.9°) [30]. Upon dynamic monitoring, women exhibited greater varus-valgus laxity than men (3.6° vs. 2.7°), with a modest positive correlation with age in both sexes [31]. During walking, women displayed 2° less varus and a lower second-peak knee-adduction and higher knee-flexion moment than men [32], the sex-differences in OA biomechanics being reviewed elsewhere [33,34].

ii. Subchondral and Subarticular Bone

**Fig. 2:**
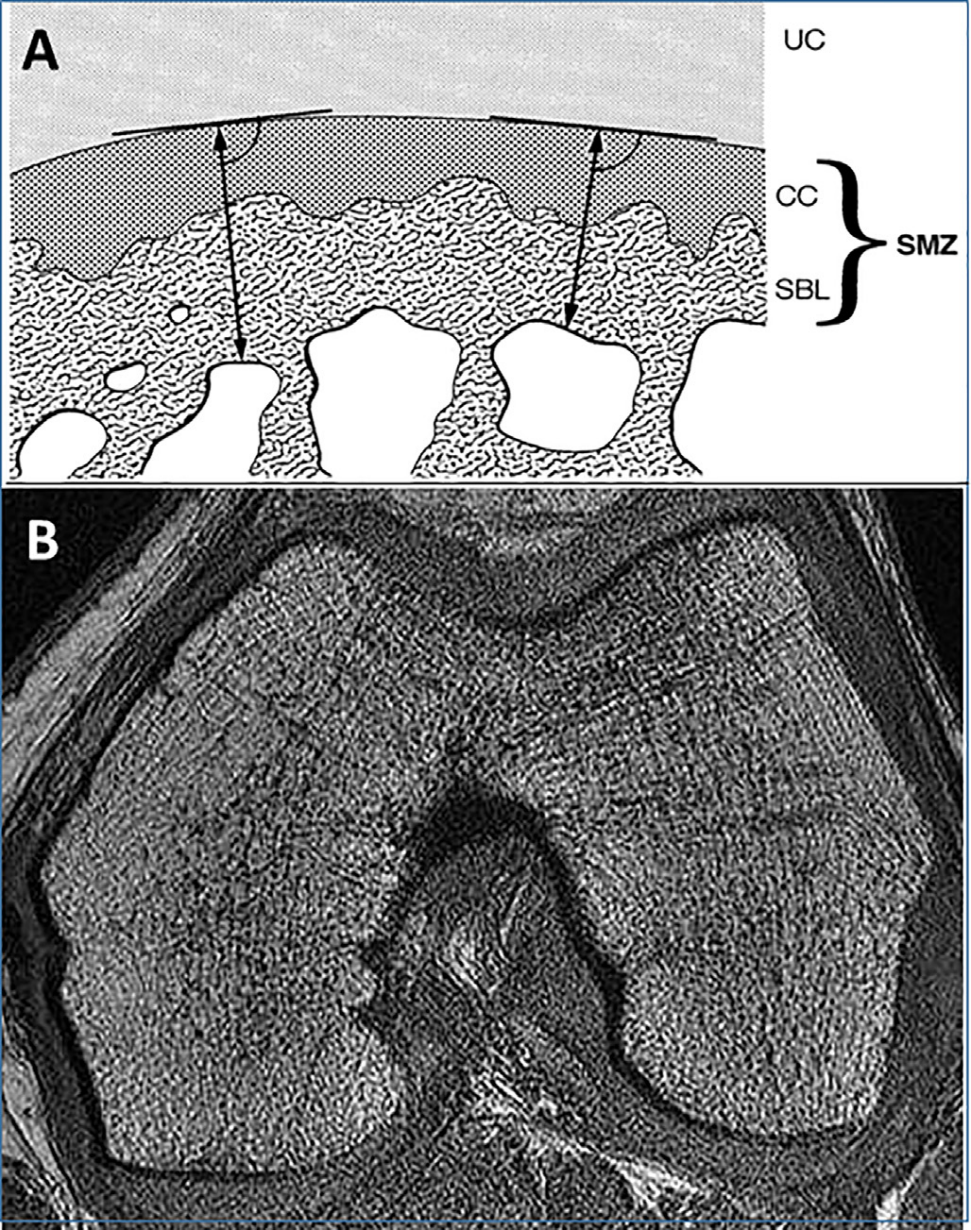
Subchondral bone (anchoring the uncalcified cartilage) and subarticular (trabecular) bone: A) The subchondral mineralized tissue zone (SMZ: ↔ arrows) composed of the calcified cartilage layer and the (cortical) subchondral bone lamella; B) Visualization of the distal femoral SMZ and subarticular trabecular bone architecture by high-resolution magnetic resonance imaging (MRI): Axial images acquired on a 1.5T GE SIGNA echo-speed system (GE Medical Systems, Waukesha, WI)) using a 3-D fast gradient-echo sequence (TE ​= ​4.5 ​ms, TR ​= ​30 ​ms, flip angle ​= ​40°, slice thickness ​= ​1 ​mm; in-plane resolution ​= ​0.195 ​mm ​× ​0.195 ​mm, FOV ​= ​10 ​cm, scan time ​= ​18:26 ​min). Image kindly provided by Gabby Joseph and Thomas Link, University of California San Francisco, CA, USA.

A 100–1500 ​ ​μm thin subchondral (cortical) bone layer [35] is bounded with the joint cartilage by a calcified layer that encompasses about 3–9% of non-calcified cartilage thickness [36]. Together these are termed “subchondral mineralization zone” (SMZ) [37] and are supported by bone trabeculae (Fig. 2a). The SMZ was found thicker in normal male than female patellar specimen (620 ​± ​120 ​μm vs. 440 ​± ​70 ​μm) [37].

**Fig. 3:**
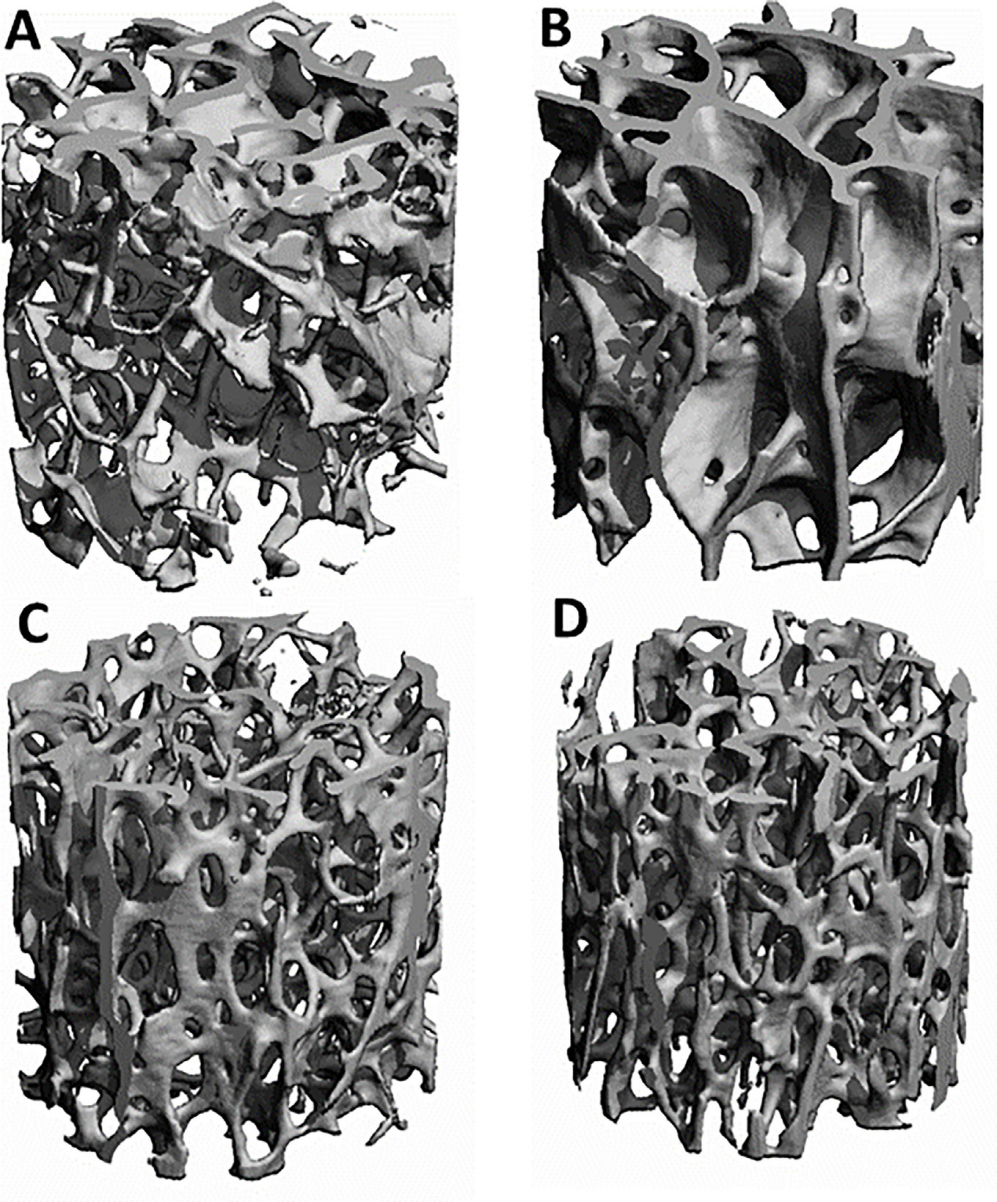
Micro-CT visualization of cylindrical trabecular bone specimen from the femoral neck and distal radius (below): A) female femoral neck; B) male femoral neck; C) female distal radius; D) male distal radius.

In children aged 6–12, trabecular bone microarchitecture at the lateral distal femur (MRI) revealed no sex-differences [38]. In older adults aged 52-99 ​y, however, sex-differences in trabecular bone micro-architecture (micro-CT) varied substantially between skeletal sites [39]: In the radius and femoral neck, trabecular bone was more plate-like, with more and thicker trabeculae, smaller separation, higher connectivity, and higher anisotropy in men than in age-matched women [39] (Fig. 3). At the calcaneus, iliac crest, and lumbar vertebrae, in contrast, no significant sex-differences were observed in (trabecular) bone volume/(total) tissue volume (BV/TV) and microarchitecture [39]. A significant age-dependent decrease in BV/TV was observed in women, whereas no age-relationship was identified in men [40]. In the proximal tibia, women exhibited a consistently lower BV/TV than men, the age-dependent decline in trabecular number being greater in women, whereas the decrease in BV/TV and trabecular thickness were similar in men and women [41]. No differences in proximal femur trabecular micro-architecture were identified between women and men in hip-OA and fragility cohorts [42]. In the hip-OA cohort, (superficial) subchondral bone showed a somewhat sclerotic micro-architecture; whereas no sex-differences were observed in the subchondral layer, these were present, however, in the deeper trabecular bone [43]. In proximal tibiae at late-stage knee-OA, trabecular thickness and number in the medial, and BV/TV in the lateral tibia were greater in women than men [44]. In radiographs, longitudinal sex-differences in projected tibial bone architecture (fractal dimension) was seen in horizontal but not in vertical trabeculae in OA (but not in non-OA) knees [45]. Cross-sectionallysex was associated with baseline vertical (rather than horizontal) fractal dimension in OA progressors, but not in non-progressors. Further, baseline medial tibial plateau fractal signature predicted medial radiographic progression [46]. In the OAI FNIH biomarker consortium sample [[47], [48], [49]], sex was significantly associated with vertical and horizontal fractal signature parameters (slope), and the summed composite of parameters obtained at three time points was associated with radiographic/symptomatic progression status [50]. The longitudinal relationship between femorotibial trabecular bone structure with cartilage morphology (and composition) change was studied in OA patients using MRI [51] (Fig. 2b) and radiographs [52], but sex-differences were not reported.

In Framingham subjects without RKOA, 54% women and 50% men had bone marrow lesions (BMLs ​= ​non-cystic subchondral areas of ill-defined high MRI signal, p ​= ​0.25) [25]. 28% women and 22% men had subchondral cysts (areas of increased subarticular MRI signal, with sharply defined rounded margins, p ​= ​0.12) [25]. These sex-differences became even less when stricter reading definitions were applied [25], and this was confirmed for BMLs in a meta-analysis of >4000 knees [26]. Sex was not found to be associated with presence, development, or persistence of BMLs over 2 years [53]. In a non-injury cohort, 13% were reported to display BMLs, these being positively and independently associated with male sex, presence of cartilage defects, tibial bone area, age, and body height [54]. Further, estradiol levels were positively associated with lower grades of BMLs [55].

iii. Synovial Tissue and Infra-patellar Fat Pad (IPFF)

**Fig. 4:**
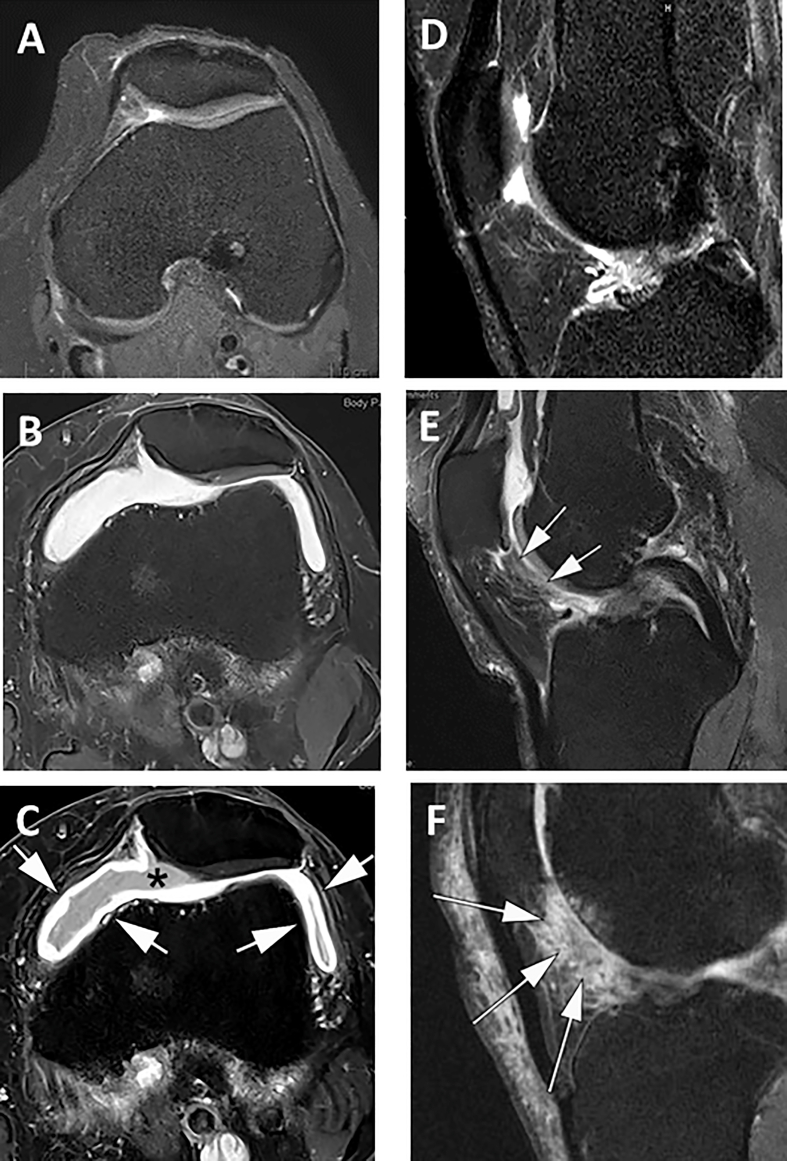
MR imaging of effusion and Hoffa synovitis: A) Axial intermediate-weighted fat suppressed non-enhanced image (3T) not showing any signs of effusion synovitis, B) Axial intermediate-weighted fat suppressed non-enhanced image (3T) depicting severe effusion-synovitis, not differentiating effusion (intra-articular joint fluid) from thickening of the synovial membrane, C) Axial T1-weighted fat suppressed image after i.v. contrast administration (3T) allowing for differentiation of the hypointense joint fluid (asterisk) and the (enhanced) thickened synovial membrane, reflecting synovial hyper-vascularization (arrows), D) Sagittal intermediate-weighted non-enhanced fat-suppressed image (3T) not showing any signs of Hoffa synovitis, E) Sagittal intermediate-weighted fat-suppressed non-enhanced image (3T) showing moderate Hoffa synovitis, most pronounced in the posterior IPFP adjacent to the synovial membrane (arrows), F) Sagittal intermediate-weighted non-enhanced fat-suppressed image (1.5T) displaying severe Hoffa synovitis with marked diffuse signal hyperintensity in the body of the IPFP (arrows). Images kindly provided by Frank Roemer, MD, Universitätsklinikum Erlangen, Germany.

The synovial membrane delineates the interior surface of the joint capsule and ligaments, secreting synovial fluid to aid joint lubrication. The term Osteo-“Arthritis” rather than “Arthrosis” stresses the (low-grade) inflammatory component, at which the synovial membrane becomes thickened, causing joint effusion (Fig. 4). It was reported that estrogen-sensitized synoviocytes with increased expression of inducible nitric oxide synthases and interleukin (IL)-1β in female rats may contribute to the greater incidence and progression of temporomandibular OA compared with male animals, an effect ameliorated by ovariectomy, blockage of estrogen receptors, or injection of estrogen receptor antagonists [56]. Further preclinical OA models pointed to sex-differences in gene expression of inflammatory molecules, hormonal receptors, and responsiveness to hormonal stimulation [57]. Men were shown to exhibit greater serum levels of hyaluronic acid (a constituent of synovium and cartilage, indicative of synovial inflammation) in OA than women, independent of age, ethnicity, BMI, OA burden, or comorbidities [58].

In the Framingham cohort, prevalence rates of effusion-synovitis (Fig. 4b and c) were similar in women (35%) and men (38%; p ​= ​0.49), but when stricter definitions were applied, women displayed less synovitis (3%) than men (6%; p ​= ​0.02) [25]. Since the study did not use contrast-enhanced MRI (Fig. 4), synovitis was considered present when the synovial cavity was distended and filled with fluid, but without ability to differentiate membrane thickening and effusion [25]. Independent of sex, effusion synovitis was associated with knee pain [59]. In women, serum levels of endogenous estradiol, progesterone and testosterone were associated with greater pain and knee effusion-synovitis volume [55]. In participants with symptomatic knee OA, estradiol, progesterone, and testosterone were inversely associated with effusion-synovitis volume in women, whereas testosterone was associated with knee pain [55]. Men displayed higher IGF-1-levels amongst cytokines in synovial fluid, but no sex-differences were noted in other biochemical markers [60]. Chemokine levels were higher in men, whereas inflammatory cytokine levels elevated in women, suggesting women to experience greater pain per level of inflammation [61,62]. Animal studies showed that aged rats, particularly females, are more vulnerable to chronic pain conditions than younger (male) rats [63].

**Fig. 5:**
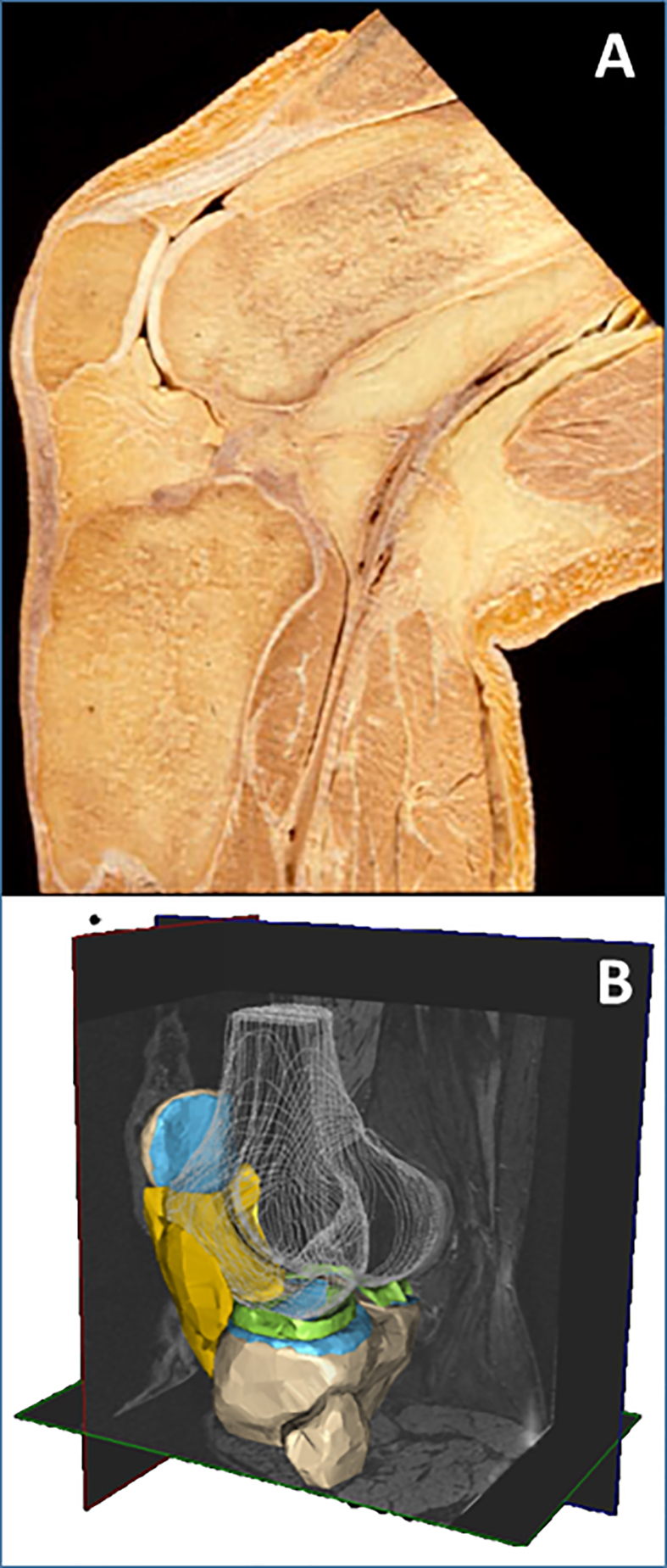
The infra-patellar fat pad (IPFP) or Hoffa: A) Sagittal anatomical section through the knee displaying the IPFP between the patella, distal femur, and proximal tibia; B) 3D reconstruction of (peri-) articular tissues of the knee from MRI showing the IPFP in yellow.

The IPFP (Hoffa) is located within the joint capsule, but outside the synovial membrane, but with immediate spatial contact to the latter (Fig. 5). Because of its intra-articular location, endocrine IPFP activity can directly affect joint structure [64,65]. Although its cellular composition is similar to that of subcutaneous fat (SCF), it produces more pro-inflammatory mediators and may represent a link between obesity, low-grade inflammation, and knee OA via endocrine pathways [65,66]. MRI signal alterations in the IPFP adjacent to the synovial membrane are regarded as Hoffa synovitis [67] (Fig. 4e and f). The role of the IPFP in knee OA, however, has also been described as protective mechanical shock absorber [66,68].

In young girls and boys aged 4-17 ​y (14–105 ​kg), IPFP volume almost linearly increased by 2 ​cm^3^ per annum [69]. The ratio of IPFP volume/body weight was greater in boys (0.54 ​cm^3^/kg) than in girls (0.45 ​cm^3^/kg); no statistically significant association of this ratio was observed with age [69]. In the healthy reference cohort of the OAI (OAI-HRC), men displayed a significantly greater (41%; p ​< ​0.001) IPFP volume, and greater IPFP volume/body weight ratio (9%; p ​< ​0.01) than women [70]. Yet, men displayed slightly (2%) more intermuscular fat (IMF) relative to the total thigh cross sectional area (CSA), and significantly less (53%; p ​< ​0.01) SCF than women [70]. The IPFP volume depended on BMI (p ​= ​0.001), with the difference relative to normal weight subjects being 10% (18% for bodyweight) in pre-obese (BMI 25–30), 17% (39%) in obese class I (BMI 30–35), and 15% (59%) in obese class II participants (BMI 35–40) [71]. The relative increase in IPFP volume compared to normal weight was not of the same magnitude nor linear with that in body weight, but results were similar for both sexes [71]. This was confirmed by reports that male sex and fat free mass were both associated with a larger IPFP in patients with RKOA [72].

In women, IPFP volume was found responsive to weight loss, but not to weight gain [73]. A significant (p ​< ​0.01) reduction in IPFP volume was observed with exercise (2.1%), diet (4.0%), and both interventions combined (5.2%) over 1.5 ​y [74], but the above studies did not stratify for sex. In an OAI sample without RKOA (POMA [75]) being overweight (BMI ≥25 and ​< ​30) and displaying Hoffa (IPFP)-synovitis significantly increased the risk or incident radiographic OA 2 ​y later in women, but not men, and the same was observed for effusion synovitis [67].

iv. Muscle and Adipose Tissue (of the Thigh)

**Fig. 6:**
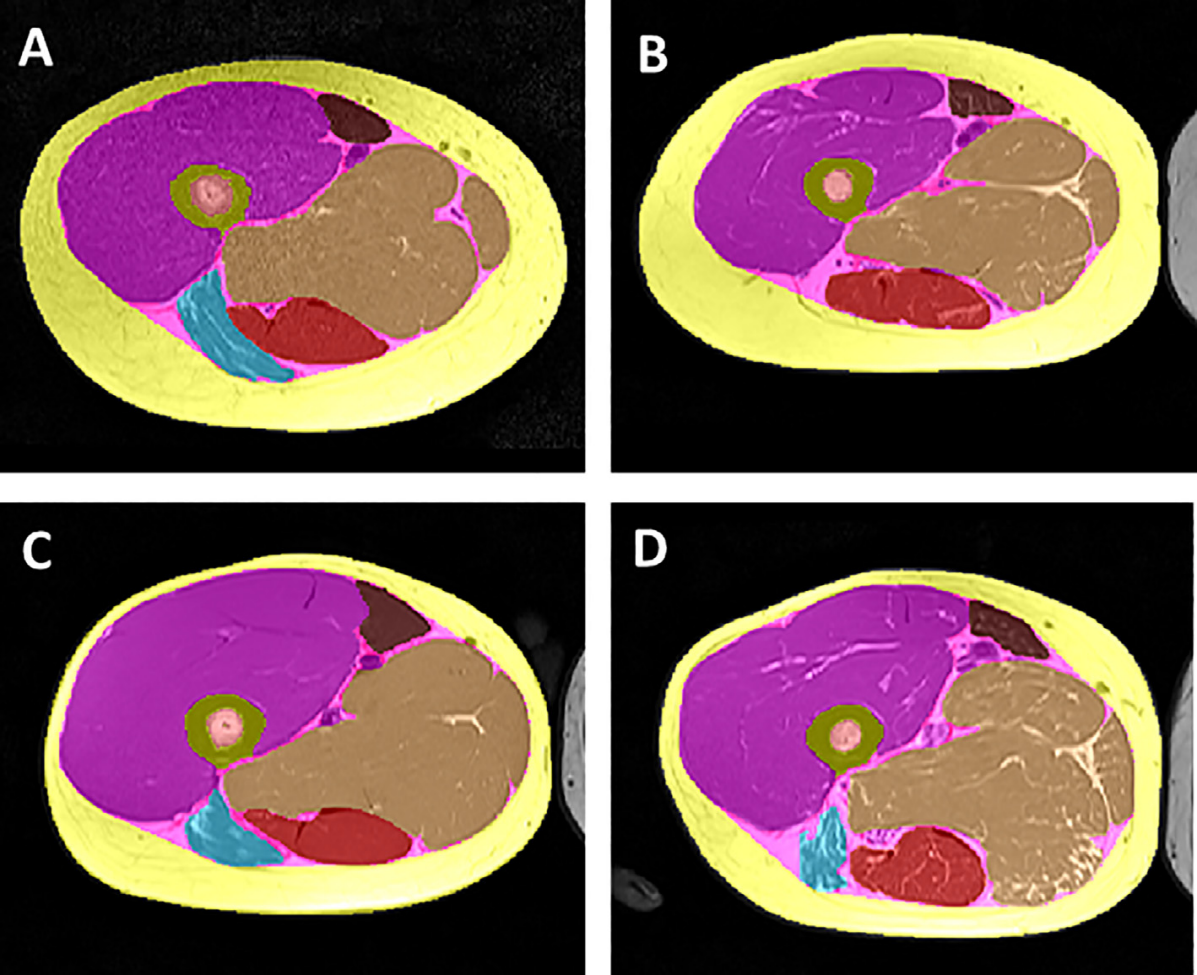
Axial MRIs of the thigh at a defined anatomical location (30% distance between femoral neck and the rectus tendon) display the quadriceps (violet) and other muscles, inter-muscular fat (IMF, pink) and subcutaneous fat (SCF, yellow): A) Adolescent female athlete; B) Mature female athlete; C) Adolescent male athlete; D) Mature male athlete.

Muscle and adipose tissue are important in joint function and OA from biomechanical and endocrine perspectives (Fig. 6). It has been recognized that amongst (thigh) adipose tissue compartments, IMF is particularly relevant to physical performance [76]. Men displayed 33% greater quadriceps muscle CSAs than women, both in adolescent and mature athletes [77]. Yet, men exhibited approximately 25% greater cortical and total bone CSA, so that the ratio quadriceps/bone CSA was only slightly greater than in women [77]. Women exhibited more SCF than men in adolescent (+77%) and mature participants (+39%), the difference increasing when normalized to bone size (+128%/+79%). Adolescent and mature women also displayed more IMF than men (+45%/+6%, when normalized to bone CSA), the sex-difference being considerably smaller than for SCF [77] (Fig. 6). Over 2 years, adolescent boys displayed statistically significant muscle (+5.0%) and bone growth (+2.9%), whereas adolescent girls did not (+0.8%/+1.9%). Adolescent and mature female athletes showed a statistically significant SCF increase (+11%/+6.0%), whereas adolescent and mature men did not [77]. Changes in IMF, SCF and muscle during longitudinal weight gain and loss failed to show obvious sex-differences in response [78]. Women were reported to display a significantly (p ​< ​0.0001) greater ratio of vastus lateralis vs. medialis (VL/VM) than men [79], but the authors used axial thigh MRIs acquired at a fixed distance proximal to the knee joint space (OAI acquisition protocol [21,22]). Given that male thighs are larger than female ones, the fixed distance involved more proximal sampling of the MRI in the (smaller) women, and more distal one in the (taller) men. As the VM extends more distally than the VL, the discordance in anatomical acquisition site may explain the observed sex-difference in VL/VM ratio [79]. Hence, care must to be taken to choose consistent sampling locations between men and women [80].

In OAI participants with discordant knee pain, the knee with frequent knee pain displayed 5.2% smaller quadriceps CSAs and 7.8% lower extensor force, these differences being similar between women and men [81]. No significant side differences in thigh muscle CSAs, strength, or specific strength divided by muscle CSA, were observed in women and men in whom one knee showed an osteophyte [82] or JSN [83] and the other did not, suggesting that (only) painful RKOA may impact muscle mass and strength. Women (but not men) with unilateral frequent knee pain displayed statistically significant greater IMF (but not SCF) CSAs, and no significant between-knee differences between the above radiographic strata [80]. In older participants, somewhat discordant findings were made using CT: those with RKOA displayed larger thigh muscle and IMF CSAs, and lower specific strength, than those without RKOA in both sexes, independent of pain status [84].

In the absence of RKOA, thigh extensor and flexor muscle specific strength were significantly associated with increased risk of incident osteophytes and JSN over 4 ​y in women, but not in men, whereas muscle CSAs were not in either sex [85]. In women, this statistically significant relationship of specific strength was lost when adjusting for BMI. Further, lower specific strength was associated with greater BMI in women (but not in men), whereas absolute strength was positively associated with BMI in men (but not in women) [85]. These findings may provide clues why women with muscle strength deficits have a poorer prognosis of OA than men with similar deficits [85]. In the same cohort, baseline waist-height-ratio, a measure of central adiposity, and anatomical thigh SCF and IMF CSAs were significantly associated with incident knee OA in both sexes [86]. Women (but not men) displayed a significantly greater reduction of knee extensor strength concurrent with incident knee pain than non-incident controls [87]. Longitudinal reduction in quadriceps CSAs in OAI participants with and without structural knee progression did not differ between both sexes [88]. However, men with progression exhibited greater longitudinal SCF CSA increases (+13%) than those without (−1.9%), whereas women with progression displayed greater IMF CSA increases (+12% vs. +1.5%) [80]. In participants of the MOST cohort [89], quadriceps weakness increased the risk of lateral femoropatellar cartilage damage worsening in women, but not in men; however, no relationship was found with medial femoropatellar or lateral femorotibial cartilage worsening in either sex [90]. Female sex, age, and BMI were positively, and ambulatory and sporting activity negatively associated with vastus medialis fat infiltration [91]. After adjusting for confounders, lesser fat infiltration was associated with reduced loss of medial tibial and patellar cartilage volume [91]. Knee extensor strength was significantly lower in female OAI participants prior to knee replacement (KR) than in non-KR controls matched by demographic factors and disease stage, whereas no such relationship was seen in men [92]. Similar results were reported for knee flexor strength, and for longitudinal change in extensor and flexor strength over 2 ​y (but not over 4 ​y) prior to KR [92]. This difference was due to a reduction in extensor muscle CSA and in specific strength over the 2 ​y prior, and directly prior, to KR in women [93].

v. Peri-articular tissue response to treatment

Pharmacologically, analgesic effects were observed to be greater in women than in men in non-OA populations [94,95]. Yet, Tanezumab, a symptomatic OA drug, was not observed to be more effective in women than in men, although women displayed more adverse events [96]. Lutikizumab (an IL-1 inhibitor thought to potentially modify joint structure) displayed no apparent effect on effusion synovitis, with only a small effect on pain in one of three doses groups [97]; however, these findings were not stratified by sex [97]. This lack of stratification applies to many other studies on the efficacy of symptom- or structure- (disease-) modifying therapy.

Non-pharmacological interventions reported differential responses [98]: 18-week resistance exercise training in older participants (>65 years) improved isometric knee extensor strength, quadriceps muscle ACSA, and muscle specific strength. Men displayed substantially greater (p ​< ​0.05) increases in muscle (specific-) strength than women (P ​< ​0.05), but the mechanisms underlying the difference remains to be established [98]. A meta-analysis of young and middle-aged subjects reported males and females to similarly adapt to resistance training, with women displaying a larger effect on relative upper-body strength gain [99]. The authors speculated that lower baseline fitness in upper-body strength in untrained women may create a greater adaptive window [99]. A recent review on integrative acute and chronic responses to exercise resumed that diversity exists across physiological responses to intervention, with sex being one of the factors in optimizing outcome [100]. Future studies of sex-specific responses to exercise were demanded to optimize training patterns, athletic excellence and health outcomes across sexes [100].

## 4 Conclusions

Osteophytes and subchondral bone attrition are equally prevalent in non-RKOA subjects. 3D bone shape differs significantly between sexes, but the OA-driven trajectory of bone shape change is similar. Static or dynamic knee alignment measures provide no compelling evidence for biomechanical causes of greater knee OA prevalence in women. Sex-differences in trabecular micro-architecture greatly depend on skeletal site and increase with age; their role in knee OA remains elusive. Presence of BMLs and subchondral cysts is similar between sexes in non-RKOA populations, and so is effusion-synovitis, albeit more frequent in men when stricter reading definitions are applied. Presence of effusion- and Hoffa-synovitis is associated with greater risk of incident knee OA in overweight women, but not in men; women appear to experience greater pain per degree of synovitis. The IPFP of men is larger than in women, also after normalization to body weight; IPFP sex-differences are greater than for IMF and smaller than for SCF. The ratio of quadriceps to femoral bone CSA is slightly greater in men, but women exhibit more IMF and SCF relative to bone CSA. Reduced thigh muscle specific strength is associated with incident RKOA in women, but not in men. Women also exhibit a greater reduction of knee extensor strength concurrent with incident pain than non-incident controls, whereas men do not. Reduction in extensor muscle CSA and specific strength also precede surgical KR in women, but not in men, and older men appear to display a greater response to resistance training than older women. These striking sex-differences may explain why women with muscle strength deficits have a poorer prognosis than men with similar deficits. Taken together, these data provide hints to the effect that female knees may be structurally disadvantaged through genuine sex-differences in peri-articular tissues morphology, particularly thigh muscle and adipose tissue properties, and therefore at greater risk of incident and progressive knee OA. Further, low-grade inflammation in obese women deserves further study, and the role of hormonal factors in sexual dimorphism of OA.

Recommendations to the reporting of sex-differences in OA research include better indexing of studies reporting sex-differences, presentation and statistical analysis of sex-specific strata, awareness of potential confounding, and need to establish sex-specific healthy reference data at various developmental and maturity stages. In terms of methodology, if the anatomical structure of interest cannot be fully covered by the measurement, care must be taken that sampling of tissue or images occurs at anatomically corresponding locations in view of men being generally taller than women. Whereas some tissue measures are independent of body height (i.e. trabecular bone architecture, specific muscle strength), others are note (i.e. bone size, IPFP volume, muscle and adipose tissue CSAs, muscle strength). In these, observed sex-differences must be differentiated from genuine ones, the latter potentially revealed by matching women and men for height, or by applying normalization. Relating IPFP volume to body weight, muscle and adipose tissue CSAs to bone CSAs, or muscle strength to muscle CSAs (specific strength) are examples for that. Relating IPFP or muscle volume to body weight may be appropriate in normal weight subjects, but problematic in obese individuals, so that analyses comparing women and men should be additionally matched for BMI. Normalization also has challenges when studying sex-differences of longitudinal tissue change [3].

Appropriate analysis, indexing, and reporting of sex-specific differences in preclinical or clinical, observational or interventional OA studies should be pursued urgently by self-commitment of the research community. More stringent rules should be gradually adopted into official editorial policies of scientific journals and text-books, and potentially also into regulatory guidance for the approval of new OA drug. Furher a strong appeal has been made that more sex-specific therapeutic research is required to optimize strategies for better clinical trials and health outcomes [4].

## Authors contributions

(1) All authors were involved in the conception and design of this review, or the selection of articles, or the analysis and interpretation of data in those articles; (2) All authors contributed to drafting the article or revising it critically for important intellectual content. The provision of the first complete draft of the article was made by Felix Eckstein; (3) All authors gave their final approval of the manuscript to be submitted.

## Responsibility for the integrity of the work

Responsibility for the work as a whole, from inception to finished article, is taken by Felix Eckstein.

## Funding and role of the funding source

No funding was received for this review and no one other than the authors or researchers in the acknowledgement had any direct or indirect influence on the selection of the content and papers presented.

## Declaration of competing interest

Felix Eckstein and Wolfgang Wirth are employees of Chondrometrics GmbH, a company that provides professional image analysis service to researchers in academia and to the pharmaceutical industry. Felix Eckstein, Wolfgang Wirth and Reinhard Putz are owners of Chondrometrics GmbH. Felix Eckstein has provided consulting services to Merck KGA, Kolon Tissue Gene, Galapagos, Novartis, 4P Pharma, and TrialSpark.

Felix Eckstein and Wolfgang Wirth have received funding from multiple sources, including public bodies and the pharmaceutical industry (detailed list upon request).
