# Supplementary material for: Sexual dimorphism in peri-articular tissue anatomy – More keys to understanding sex-differences in osteoarthritis?
Source: Osteoarthr Cartil Open. 2024 May 11;6(3):100485. doi: 10.1016/j.ocarto.2024.100485 (PMC11214405; doi:10.1016/j.ocarto.2024.100485)
Supplement: Multimedia component 1 [file mmc1.pdf]

Osteoarthritis and Cartilage

# Sexual Dimorphism in Articular Tissue Anatomy – Key to Understanding Sex-differences in Osteoarthritis?

--Manuscript Draft--

| | |
|---|---|
| Manuscript Number: | |
| Article Type: | Special Issue: Sex Differences in OA |
| Section/Category: | Clinical |
| Keywords: | Articular Tissue; Sex; Joint; knee; Osteoarthritis |
| Corresponding Author: | Wolfgang Wirth, Ph.D.<br>Paracelsus Medical Private University Institute for Anatomy and Cell Biology<br>Salzburg, AUSTRIA |
| First Author: | Felix Eckstein |
| Order of Authors: | Felix Eckstein |
| | Wolfgang Wirth, Ph.D. |
| | Reinhard Putz |
| Abstract: | Objective: Osteoarthritis (OA) prevalence/incidence varies between women and men, but it is unknown whether this follows sex-specific differences in the systemic disease process (e.g. hormones), and/or differences in pre-morbid joint anatomy. We recognize that classifications of sex within humans cannot be reduced to female/male, but given the lack of literature on non-binary individuals, this review is limited to the sexual dimorphism of articular tissues.<br>Methods: Based on a Pubmed search and input from experts, we selected relevant articles based on subjective judgement of originality, relevance, and interest; no objective, bibliometric measures were used to evaluate the quality or importance of the work. Focus was on clinical rather than pre-clinical studies, with most (imaging) data being available for the knee joint.<br>Results: After introducing sexual dimorphism, specific literature of articular entities is reviewed: 1) radiographic joint space width (JSW), 2) meniscus and ligaments; 3) cartilage metrics; 4) cartilage composition and deformation; 5) articular tissue response to treatment.<br>Conclusions: Clear sex-specific differences were observed for JSW, meniscus damage, ligament properties, and cartilage metrics (volume, thickness and surface areas), but not for cartilage composition. Cartilage metrics were smaller in women even after matching for body height, weight, and age. Taken together, the findings indicate that female (knee) joints may be structurally more vulnerable and at greater risk of OA. The "one size/sex fits all" approach must be abandoned in OA research, and all observational and interventional studies should report their results for sex-specific strata, at least in pre-specified secondary or post-hoc analyses. |

Powered by Editorial Manager® and ProduXion Manager® from Aries Systems Corporation

Click here to access/download;Cover Letter;Cover Letter - OAC Sex Differences in Osteoarthritis.doc

PARACELSUS
MEDIZINISCHE PRIVATUNIVERSITÄT

INSTITUTE OF ANATOMY
Director: Univ. Prof. Dr. Felix Eckstein

Salzburg, November 14, 2023

**A.M. Malfait, MD, PhD & D. Hunter MD, PhD**
**Editors in Chief**
**Osteoarthritis & Cartilage**

Dear Drs Malfait and Hunter,

Dear Dieuwke and Dr. Maerz,

we hereby submit the review previously discussed for the special issue of OAC on sex differences in osteoarthritis:

**Sexual Dimorphism in Articular Tissue Anatomy – Key to Understanding Sex-differences in Osteoarthritis? by Felix Eckstein, Wolfgang Wirth and Reinhard Putz**

We have structured the review into Introduction, Methods, Results and Conclusion/Future Perspective, with the results containing five chapters:

1) Radiographic JSW,

2) Meniscus & Ligaments,

3) Articular Cartilage Metrics,

4) Articular Cartilage Composition and Deformation,

5) Articular tissue response to treatment

It was not possible to cover all tissues in this article as this would have by far exceeded the allowed word count. We have therefore limited the review article to articular tissues only and plan to submit the part covering peri-articular tissues separately at a later point. The length of the manuscript still exceeds the word count by 10% and we hope that this is acceptable as discussed via email.

Strubergasse 21 · A-5020 Salzburg · Internet: www.pmu.ac.at · Tel.: +43 (0)662 / 44 2002-1240 · Fax: +43 (0)662 / 44 2002-1249
Rechtsträger: Paracelsus Medizinische Privatuniversität Salzburg - Privatstiftung · FN 191581m · Landesgericht Salzburg · DVR: 103 2470

PARACELSUS
MEDIZINISCHE PRIVATUNIVERSITÄT

INSTITUTE OF ANATOMY
Director: Univ. Prof. Dr. Felix Eckstein

We hope you like the review article and find it suitable for the special issue of OAC. We would be very proud and honored being able to contribute to it.

Very warm regards

Univ. Prof. Dr. med. Felix Eckstein

Strubergasse 21 · A-5020 Salzburg · Internet: www.pmu.ac.at · Tel.: +43 (0)662 / 44 2002-1240 · Fax: +43 (0)662 / 44 2002-1249
Rechtsträger: Paracelsus Medizinische Privatuniversität Salzburg - Privatstiftung · FN 191581m · Landesgericht Salzburg · DVR: 103 2470

# Sexual Dimorphism in Articular Tissue Anatomy – Key to Understanding Sex-differences in Osteoarthritis?

**Felix Eckstein[1,2,3], Wolfgang Wirth[1,2,3], Reinhard Putz[4]**

[1] Department of Imaging & Functional Musculoskeletal Research, Institute of Anatomy & Cell Biology, Paracelsus Medical University, Salzburg, Austria

[2] Ludwig Boltzmann Institute for Arthritis and Rehabilitation (LBIAR), Paracelsus Medical University, Salzburg, Austria

[3] Chondrometrics GmbH, Freilassing, Germany

[4] Anatomische Anstalt, Ludwig Maximilians Universität München, Munich, Germany

**Running head:** Sex-differences of joint tissue

**Article Type:** @ Dieuwke Schiphof: d.schiphof@erasmusmc.nl

Special Issue / Commissioned Article / Narrative Review

**Main text word count:** 4398

**Abstract word count:** 249

**References:** 98

**Correspondence address:**

Univ. Prof. Dr. med. Felix Eckstein, Center of Anatomy & Cell Biology, Paracelsus Medical University, Strubergasse 21, 5020 Salzburg Austria; E-mail: felix.eckstein@pmu.ac.at

**Conflict of Interest (COI) Statement:**

Felix Eckstein and Wolfgang Wirth are employees of Chondrometrics GmbH, a company that provides professional image analysis service to researchers in academia and to the pharmaceutical industry. Felix Eckstein, Wolfgang Wirth and Reinhard Putz are owners of Chondrometrics GmbH. Felix Eckstein has provided consulting services to Merck KGA, Tissue Gene, Galapagos, Novartis, 4P Pharma, and TrialSpark. Felix Eckstein and Wolfgang Wirth have received funding from multiple sources, including public bodies and the pharmaceutical industry (detailed list upon request).

**Keywords:**

- Articular Tissue
- Sex
- Joint
- Knee
- Osteoarthritis

**Authors Contributions**

(1) All authors were involved in the conception and design of this review, or the selection of articles, or the analysis and interpretation of data in those articles.

(2) All authors contributed to drafting the article or revising it critically for important intellectual content. The provision of the first complete draft of the article was made by Felix Eckstein.

(3) All authors gave their final approval of the manuscript to be submitted.

**Responsibility For the Integrity of the Work**

Responsibility for the work as a whole, from inception to finished article, is taken by Felix Eckstein

**Funding and Role of the Funding Source:**

No funding was received for this review and no one other than the authors or researchers in the acknowledgment had any direct or indirect influence on the selection of the content and papers presented.

**ABSTRACT**

**Objective:** Osteoarthritis (OA) prevalence/incidence varies between women and men, but it is unknown whether this follows sex-specific differences in the systemic disease process (e.g. hormones), and/or differences in pre-morbid joint anatomy. We recognize that classifications of sex within humans cannot be reduced to female/male, but given the lack of literature on non-binary individuals, this review is limited to the sexual dimorphism of articular tissues.

**Methods:** Based on a Pubmed search and input from experts, we selected relevant articles based on subjective judgement of originality, relevance, and interest; no objective, bibliometric measures were used to evaluate the quality or importance of the work. Focus was on clinical rather than pre-clinical studies, with most (imaging) data being available for the knee joint.

**Results:** After introducing sexual dimorphism, specific literature of articular entities is reviewed: 1) radiographic joint space width (JSW), 2) meniscus and ligaments; 3) cartilage metrics; 4) cartilage composition and deformation; 5) articular tissue response to treatment.

**Conclusions:** Clear sex-specific differences were observed for JSW, meniscus damage, ligament properties, and cartilage metrics (volume, thickness and surface areas), but not for cartilage composition. Cartilage metrics were smaller in women even after matching for body height, weight, and age. Taken together, the findings indicate that female (knee) joints may be structurally more vulnerable and at greater risk of OA. The “one size/sex fits all” approach must be abandoned in OA research,

and all observational and interventional studies should report their results for sex-specific strata, at least in pre-specified secondary or post-hoc analyses.

Manuscript

Click here to view linked References

Click here to access/download;Manuscript;Review_Sex_Artonly_Tissue_2023



## Introduction

Species display a wide variety of phenotypes and inter-subject differences in general tissue anatomy and pathology. Yet, humans exhibit relatively high evolutionary homogeneity compared with other species, as the genetic repertoire originates from an African population of <1500 breeding individuals[1]. This "bottleneck" in evolution even lasted for >100.000 years, bringing human ancestors close to extinction[1].

Inter-individual differences in humans obviously depend on race, age, and many other factors, with the observed variability clustering around two "types", that is female and male sex. Classifications of human sex cannot be reduced to female/male, but given a lack of specific literature on non-binary individuals, this review article is limited to the analysis of sexual dimorphism. Since we consider biological and not sociological dimorphism, the term "sex" will be used rather than "gender".

Sex-differences vary between species: The "triplewart seadevil", for instance, is known for extreme dimorphism[2]; males (1-3 cm length) permanently attach to their female counterparts (20-30 cm) and become dependent on their blood-supply and nutrition[2]. Only a small degree of sexual dimorphism, in contrast, is found in spotted hyenas; females require high energy per litter and encounter tough competition for food, displaying similar phenotype and behavior as their male counterparts[3].

Clinical epidemiology has revealed heterogeneous findings on the prevalence, incidence, and severity of sex-differences in osteoarthritis (OA), depending on age, population, method (radiographic or symptomatic), and thresholds used to define OA. Yet, women generally demonstrate more severe clinical manifestations of musculoskeletal disorders, the inter-sex-disparity increasing with age[4]. The prevalence

of frailty, a state of increased vulnerability to stressors due to reduced physiological reserve, is more frequent in osteoarthritic women than men[5]. Women also exhibit greater OA severity and disability[6]. Knee and hand OA are more prevalent than hip OA, and more prevalent in women than in men, particularly when presenting as symptomatic[7]. Risk of incident knee and hand OA increases rapidly at age 50-75, in women more so than in men[7]. The prevalence of radiographic knee OA (RKOA) in a Chinese population was twice that in women compared with men, and symptomatic knee OA occurred 3x as often[8]. Chinese women had a 1.5x greater RKOA prevalence than those in the Framingham cohort, a mainly Caucasian population (MA, US)[9], whereas RKOA prevalence in Chinese men was similar to Framingham[8]. Hand OA (assessed in another US cohort) was only slightly more prevalent in women, but substantially more frequent than in men when being symptomatic[10]. However, no significant sex-differences were observed in the prevalence of hip OA in a large multi-continental meta-analysis[11].

It is currently unknown to what extent such sex-differences in OA epidemiology originate from discordant systemic disease processes (i.e. hormonal factors[12–14]), or from potential sexual dimorphism in joint anatomy, potentially exposing women to greater vulnerability (as also discussed for biomechanical vs. biological impact[15]). Risk factors of knee OA were shown to differ between sexes: High body mass index (BMI), alcohol consumption, atherosclerosis, and high vitamin E levels in women vs. high physical activity, soft drink consumption, and abdominal obesity in men[16]. In a meta-analysis of sex-differences in OA, the authors concluded that these have been understated in terms of risk factors[5]. Another review focusing on

MRI morphometry and systemic biomarkers of joint metabolism in OA stressed the need of studying children, adolescents and adults, before the actual onset of disease, to avoid questions of temporality in cross-sectional studies, to expose mechanisms behind sex-differences, to evaluate the predictive value of findings in OA development, to identify subjects at risk prior to disease onset, and to provide opportunities for prevention and early intervention[17]. In the current review we will thus highlight findings in healthy, pre-morbid subjects at various developmental stages.

Sex-differences in OA animal models have also been reported[13,18–20], but this review will focus on the sexual dimorphism of human joint anatomy. Whilst its scope is limited to articular tissues, we acknowledge sex-differences in other structures, for instance bone, synovial tissue, infrapatellar fat pad, peripheral muscle and adipose tissue. Due to space limitations, peri-articular tissues will be covered in a separate review.

**Methods**

We performed a Pubmed search, using a variety of search terms, including (but not limited to): sex, gender, dimorphism, joint, articular tissue, knee, meniscus, ligament, cartilage, metrics, and composition. We further contacted several experts in the field, asking them to identify and help with the interpretation of relevant literature. Articles were included/excluded based on subjective judgement of their originality, relevance and interest; no objective, bibliometric measure was used to evaluate the quality or importance of the work. Focus was on clinical studies, with most (imaging) data

available for the knee (Fig. 1). Pre-clinical studies (in vitro or in vivo) were deprioritized.

**Results**

i. Radiographic JSW

Radiographic JSW represents the radio-transparent gap between opposite bone interfaces of a joint (Fig. 2). In the femorotibial articulation, JSW is typically determined from weight-bearing, semi-flexed radiographs of proper alignment with the tibial plateau[21,22]. The medial minimum femorotibial JSW (mmJSW) has been widely used as a one-dimensional projectional metric for capturing knee OA progression[23], and has traditionally been recommended by the Food and Drug administration for evaluating treatment response to disease modifying OA drugs (DMOAD)[24]. mmJSW often is also used as entrance criterion for DMOAD trials[25], to warrant a dynamic window for measuring JSW reduction over time, without encountering floor effects. Selecting participants above a certain JSW threshold may, however, favor men, as they may exhibit greater mmJSW and body height than women. In healthy men, the mmJSW was 19% larger (5.7±0.8mm) than in healthy women (4.8±0.7mm), no significant (cross-sectional) decrease with age in either sex being noted[26]. A comparison of JSW between sexes must, of course, account for RKOA severity, as the JSW becomes smaller with greater structural involvement (Fig. 2). To this end we examined healthy reference cohort (HRC) as well as incidence/progression cohort subjects from the Osteoarthritis Initiative (OAI) with various degrees of RKOA. The OAI is a multi-center, longitudinal, prospective observational knee OA study

following almost 5000 participants for >8 years[2728]. The OAI-HRC included subjects without symptomatic or radiographic knee OA in both knees, or risk factors. Here we found the mmJSW to be 10% less than in the above study[26], but the 18% (0.8mm) difference between men and women was similar, and so was the 17% (0.8mm) difference in incidence/progression cohort knees without RKOA[26] (Fig. 3). Yet, sex-differences became less with greater joint space narrowing (JSN[29]) grade (Fig. 2) and disappeared at the highest of three JSN grades (Fig. 3). Whether this insinuates greater tissue pathology in men at late-stage disease, or is an effect of the specific reading system, remains to be determined. Yet, systematic sex-differences in JSW at early disease stage have to be taken into account when using mmJSW diagnostically, for study inclusion/exclusion, or as structural outcome in clinical studies. Sex-specific contributions of meniscus and cartilage to mmJSW are outlined in the next chapter[30].

ii. Meniscus & Ligaments

The menisci are semilunar fibrocartilaginous discs, located between the proximal tibia and distal femur (Fig. 1d; 4). They alleviate femorotibial incongruity and permit to transfer loads relatively evenly from one bone to the other, at different knee positions. 3D quantitative MRI (qMRI) meniscal metrics were studied in the OAI-HRC, after excluding knees with meniscus lesions[31]. The tibial plateau was significantly larger in men than women (+23% medially; +28% laterally; both $p<0.001$), as was the meniscus surface area. The ratio of meniscus versus tibial plateau area, and the percent tibial coverage by the meniscus (50% medially; 58% laterally) were similar between sexes. Only "physiological" medial meniscal extrusion was significantly greater (+48%;

p=0.01) in women than in men (1.8 vs. 1.2mm)[31]. Given a significant relationship between extrusion and knee pain[32], this finding may explain why women encounter symptomatic knee OA more frequently than men. The contribution of 3D meniscus vs. cartilage measures to JSW was also studied in the OAI-HRC[30]: In men, cartilage thickness correlated more strongly with JSW ($r^2$=44%) than meniscus or demographic measures; in women, meniscal and cartilage metrics displayed similar strength ($r^2$=17%)[30]. How meniscus and cartilage contribute to longitudinal change in JSW was explored[33], but sex-differences were not reported.

Englund et al.[34] studied the menisci of almost 1000 participants of the Framingham cohort, using an established radiological reading system of MRI structural pathology (WORMS[35]): Prevalence of meniscal damage increased with age from 19%/32% (women/men) in those 50-59y, to 51%/56% in those 70-90y; the sex-difference became less with age[34]. Men displayed higher prevalence of tears, but women higher prevalence of severe meniscal destruction, particularly at older age. In women (but not in men), prevalence of meniscal tears was associated with a greater BMI[34]. In Framingham subjects without RKOA, Guermazi et al.[36] reported substantially higher prevalence of meniscus damage in men than in women (35% vs. 15%; p<0.001), also when using stricter reading criteria (WORMS≥2; 12% vs. 5%; p<0.001)[36]. Meniscus extrusion was similar (2.7mm medially, 1.8mm laterally) in men and women[37], yet, the proportion of knees with medial extrusion >5mm was 7% in men, and only 2.5% in women. Another study reported very mild meniscal abnormalities (WORMS=1) to be more common in women, whilst more severe lesions

(≥4) were more common in men, with lesion prevalence increasing with higher self-reported physical activity levels[38].

In terms of MRI tissue composition (explanation see below), the meniscus MRI transverse relaxation time T2 was significantly elevated in the outer meniscal zones in women compared with men, while the inner zone did not exhibit significant sex-differences. Lateral meniscus T1ρ was slightly longer in young healthy women than men ($p=0.045$), but no sex-differences were observed in middle-aged subjects, without RKOA, or between these three groups medially[39].

Structural and biomechanical properties of ligaments (Fig. 4) and tendons differ between women and men, likely contributing to more frequent injuries in female athletes[40,41]. In the incidence subcohort of the OAI, ACL lesions were most common, followed by the patellar ligament, with ligamentous abnormalities being more prevalent in men (23%) than women (13%)[38]. Again, lesion prevalence increased with higher self-reported physical activity levels[38].

### iii. Articular Cartilage Metrics

Articular cartilage covers the bone epiphyses (Fig. 1d, 4), to provide almost frictionless gliding joint surfaces that transfer high (dynamic) loads to the (subchondral) bone, without undergoing damage for decades. These exceptional mechanical properties originate from the ability of the cartilage matrix to bind fluid, and from making use of the principle of hydrostatic pressurization, to transfer load evenly at minimal friction[42]. Healthy boys aged 9-18y were found to display greater cartilage volume than girls at this age[43], and displayed larger cartilage volume accrual

rates longitudinally (1.6 years) [44]. In both sexes, cartilage volume accrual in the tibia (but not patella) correlated with height, but not weight change[44]. Yet, cartilage volume change does not represent thickness change directly, because volume is strongly determined by metaphyseal bone growth, which may differ between girls and boys. Using ultrasound in healthy children (7-16y), boys exhibited significantly thicker cartilage than girls[45], cartilage thickness decreasing with age in both sexes[45], likely due to epiphyseal mineralization advancing towards the joint surface. In adolescent (age 16.0±0.6y) and mature (age 46.3±4.7y) athletes, men displayed 20% thicker femorotibial cartilage than women in both groups. During 2y follow-up, the annual increase in femorotibial cartilage thickness was 0.8% in adolescent boys and 1.4% in girls, without significant sex-differences[46], and with similar findings in patellar cartilage[47]. Observations in the KANON ACL cohort suggest that the increase in femorotibial[48], but not femoropatellar[49], cartilage continues well beyond age 30, similar to accrural of peak bone mass, and does not differ between both sexes[48].

Cicuttini et al.[50] first reported adult men to display significantly greater knee cartilage volume than women, the difference remaining statistically significant after adjusting for age, height, weight and bone volume. In a larger cohort, men displayed 33-42% greater knee cartilage volume than women, the magnitude of sex-differences decreasing to 8-18% after adjusting for body height, weight and bone size; further adjustment for physical activity had no effect[51]. A study in young adults reported that sex-differences in knee cartilage volume decreased after adjustment for lean body mass (38%), fat mass (20%), and plasma fibrinogen (37%)[52]. Total body bone mineral mass (BMC) and density (BMD) were significantly associated with tibial cartilage

volume, the association being stronger in men than women[53]. In healthy older men, tibial cartilage volume was inversely associated with age, BMI, and physical activity, and positively associated with BMC and serum testosterone[54]. Longitudinal reduction of tibial cartilage volume was associated with serum free testosterone (partial $r^2$=15%), independently of age, BMI, and total BMC[55].

In healthy non-athletic adults, women displayed substantially smaller cartilage volume (-20% to -47%) and joint surface areas (-21% to -33%) than men, whereas the sex-difference in cartilage thickness was much smaller (-2.0% to -13%)[56]. Similar observations were made in the ankle and subtalar joints[57]. In the knee, adjustment for height and weight reduced, but did not eliminate sex-differences in knee cartilage metrics; surface area was a strong predictor of cartilage volume (independent of sex, height and weight), but did not correlate significantly with cartilage thickness[58]. The authors further explored whether anthropometric factors from routine examination can be used to estimate pre-morbid knee cartilage metrics, to estimate cartilage loss retrospectively. The close dependence of cartilage volume on surface areas was found to subsume dependency on sex, height, weight and BMI[59]. In women, volume depended on body height cubed, and joint surface area on height squared; these relationships were much weaker in men[59]. The absence of a tight relationship between cartilage volume (and joint area size) with height in men suggests that factors stimulating bone and cartilage growth differ between sexes[59]. The high correlation between cartilage volume and joint surface / subchondral bone areas across women and men suggest that, opposite to height and weight, subchondral bone areas provide reasonable estimates of pre-morbid cartilage volume[59].

Since adjustment for confounders and allometric modeling have inherent limitations, we used OAI data to match women and men (1:1) with the same body height (±1 cm), BMI (±2), and age (±5 years), to answer whether genuine sex-differences in cartilage metrics exist, independent of height (and weight) differences. Of 1113 OAI participants (OAI-HRC or incident cohort) that did not have RKOA, 767 had qMRI cartilage measurements; of those, 63 pairs could be precisely matched based on the above criteria. Men exhibited 23% greater femorotibial cartilage volume, 10% greater joint surfaces, and 11% greater cartilage thickness, and altough these differences were smaller than those in non-matched individuals (see above), differences were highly statistically significant ($p<0.001$).

Sex-specific cartilage thickness reference values throughout 16 commonly used femorotibial subregions were reported for Framingham and OAI-HRC[60] cohorts (Fig. 5), suggesting that men display thicker cartilage anywhere, but similar maximal Z-scores as women, due to greater thickness variability. A report of reference values was extended to that of subchondral bone area and to various radiographic stages in >1000 OAI participants[61]. Sex-differences in knee cartilage metrics appeared to become greater at age >50, indicating that both cartilage development in early and cartilage loss in later life contribute to these differences[51].

Examining whether joints functionally adapt to increased mechanical loading, neither cartilage volume nor thickness differed significantly between female or male thriathletes vs. physically inactive volunteers, but knee joint surface areas were significantly greater in male (+8.8%; $p<0.01$) and non-significantly greater in female (+7.0%) triathletes than in their inactive counterparts[62]. The results suggest that

functional adaptation may occur to more frequent loading, at the level of the joint size, but not in cartilage thickness. This effect may be somewhat more pronounced in men than women[62]. 4y cartilage thickness change was related to the Physical Activity Scale for the Elderly (PASE) score in OAI participants with RKOA: Women with low physical activity displayed greater cartilage loss than those with moderate physical activity, but no significant difference between activity groups was observed in men[63].

Amongst non-RKOA Framingham subjects, prevalence of cartilage lesions was similar in men and women (69% vs. 70%), also when using stricter definitions (44 vs. 43%)[36]. Yet, studying knees with RKOA from the MOST cohort, men with Kellegren-Lawrence-grade (KLG) 2 displayed MRI cartilage lesions more often than women in the medial femorotibial compartment (88% vs. 75%), but less often in the lateral femorotibial (49% vs. 62%) and femoropatellar compartment (77% vs. 86%)[64]. In KLG3 participants, only in the femoropatellar compartment women were significantly more affected than men (93% vs. 80%)[64]. In the OAI incidence cohort, cartilage abnormalities were slightly more prevalent in women than in men (77% vs 72%), mostly in the patella[38]. Full-thickness defects were more frequent in men than in women (24% vs 15%)[38].

Male knees more likely exhibited qMRI-detected fully denuded areas of (subchondral) bone (dABs) than female knees ($p<0.001$)[65]. Greater presence of dABs was related to greater RKOA grades in both men and women, but dAB size was not related to sex, age, or BMI[65]. Although dABs were related to subsequent structural progression[66] and incident knee pain[67], analyses did not report sex-differences in these relationships.

In the OAI, femorotibial (subregional) cartilage loss[61,68] over 1y depended on RKOA stage and pain, but not on age, BMI and sex. Yet, in an Australian cohort with longer (2y) follow-up, women appeared to display greater tibial cartilage volume loss than men, sex-differences appearing from age 40[69]. However, in this study, the crude annual percent volume change did not differ significantly between sexes, but the difference only became significant after adjusting for baseline cartilage volume. In a similar study, female sex appeared associated with increased risk of progression of tibial cartilage defects[70]. Risk factors associated with femoral cartilage loss were female sex, age, smoking, and reduced lower limb muscle strength[71]. Patellar cartilage loss was found unrelated to medial or lateral femorotibial cartilage loss, but also was observed to be greater in women than men[72]. Other risk factors associated with patellar cartilage loss and defects included a high BMI, waist circumference, and fat mass, particularly in women[73]. In a study over >10y follow-up, sex-differences in longitudinal cartilage loss were most pronounced in the lateral tibia, and increased with age[74].

As OA incidence increases after menopause, the role of estrogen is under investigation, and whether use of estrogen replacement therapy (ERT) is protective[14,75]. In healthy women, free and total testosterone, pre-androgens and sex hormone binding globulin levels were not significantly associated with knee cartilage volume[76], but progesterone was correlated with cartilage volume in women with symptomatic knee OA[77]. A cross-sectional study suggested that the greater knee cartilage volume loss with obesity and female sex was related to leptin, and thus may be hormonally mediated in older adults[77]. In a cross-sectional study, 81

postmenopausal women with >5y ERT appeared to display 8% greater tibial cartilage volume than non-users, independent of RKOA status[78]. This relationship was, however, not seen in partellar cartilage in the same cohort[79]. In women aged 50-80y, parity was associated with less knee cartilage volume, but the use of ERT and/or and oral contraceptives was not[80]. In a longitudinal RCT in healthy post-menopausal women, tibial cartilage loss was not modified by ERT[81]. For further work on sex hormones and structural (cartilage) changes in OA, see the following review[82].

iv. Articular Cartilage Composition and Deformation

qMRI can also be applied to examining cartilage matrix composition[83,84]. Cartilage transverse relaxation time (T2) has been found to be associated with collagen content, collagen orientation, hydration, cartilage mechanical properties, and with early OA status[84], higher values (longer T2) indicating matrix pathology. Mosher et al.[85] did not identify sex-differences in cartilage T2 between young adults. In children and adolescents, no sex-differences in T2 were noted in those with open physis, but in those with a closed (or closing) physis, T2 was significantly elevated in boys vs. girls ($p<0.05$)[86]. A longitudinal decrease over 2y in deep medial femorotibial cartilage T2 was confirmed in female and male athletic adolescents, whereas in the superficial layer, no significant longitudinal T2 change occured in either sex[87]. In adult athletes, no significant longitudinal T2 change was observed in either layer or sex[87]. Also, no sex-differences were present in patellar T2 change in adolescents, but mature male athletes displayed a statisically significant T2 increase over time in superficial and deep layers[47], which was significantly greater than in mature females.

Sex-specific bulk T2 reference values were presented in OAI participants without definite RKOA without differentiating the superficial and deep lamina: women displayed slightly greater T2 than men in most compartments, but the difference was only statistically significant in the medial femur ($p<0.0001$)[88]. Sex-specific reference values across 16 femorotibial subregions were provided for superficial and deep T2 in the OAI-HRC[89] (Figs. 6a,b). When averaged across the entire joint surface, T2 did not differ significantly between women and men in superficial or deep layers. Medially, superficial T2 tended to be longer in men than in women, and laterally, deep T2 longer in women than men; the strongest sex-differences were observed in the superficial anterior medial tibia, and in the deep internal lateral tibia and femur (women>men)(Fig. 6a). T1ρ relaxation represents another qMRI cartilage composition metric, at which greater values indicate loss of proteoglycan, and possibly concomitant increase in hydration. Middle-aged women with RKOA displayed greater ($p=0.017$) lateral femorotibial and femoropatellar T1ρ than men at these locations[39]; no sex-differences were observed in other groups (see above) or in the medial compartment[39].

In vivo cartilage deformation was studied before and after 30 deep knee bends in subjects aged 50-78y[90], scanning the knee first after ≥45 mins of rest, and then again immediately after the excercise. The difference in patellar cartilage volume (deformation) was -2.6±1.7% in older women and -2.2±1.7% in older men, these values being significantly ($P<0.05$) lower than those in young (22-30y) female (4.5±1.3%) and male participants (6.2±2.1%, Fig. 7). Yet, sex-differences did not

reach significance in either the young or older group[90]. A lack of sex-differences in femorotibial cartilage deformation also was repored after running 5000 steps[91].

v. Articular tissue response to treatment

Analgesic effects in women appear to be greater than in men[92,93], with a distinct sexual dimorphism reported in the interplay of nalbuphine and naproxen in post-operative analgesia[94]. In a potential DMOADs RCT, intra-articular sprifermin increased cartilage thickness significantly (and dose-dependently) over placebo for up to 5 years[25,95], but no stratified results for men and women were presented[25]. Surgical knee joint distraction (KJD) substantially increased cartilage thickness and radiographic JSW, with male sex (and more severe baseline RKOA status) being predictive of a greater JSW increase over 1y after KJD[96]. 10y joint survival after KJD was greater in men than in women; the increase in cartilage thickness and JSW at 1y was positively related to joint survival in men and women analyzed together[97]. Despite acknowledgement of sex-differences in cartilage biology in the scientific community, some authors argued that implications for clinical trial design remain unclear, with many studies arbitrarily assigning demographics[98]. Stem cell donor and recipient sex were proposed to contribute to sex-differences in treatment efficacy[98].

**Conclusions and future perspective:**

Using mmJSW for clinical trial inclusion, sex-specific thresholds should be used, to avoid a sex-specific selection bias. mmJSW dominantly reflects cartilage thickness in men, but both meniscus and cartilage metrics in women, a potentially undesired effect

when mmJSW is used as outcome measure for assessing structural progression. Women exhibit greater physiological meniscus extrusion, potentially contributing to greater prevalence of symptomatic knee OA in females. Cartilage volume, thickness, and surfaces areas were greater in men than in women, even after matching for height and weight. Assuming joint loads scale with body weight, this implies that mechanical load is distributed over a smaller joint surface area, leading to greater mechanical stresses in female joints. Whether women encounter greater rates of cartilage loss than men is still an open question, given methodological questions of studying relative (%) vs. absolute change, and adjustment for baseline values. The impact of sex hormones on cartilage (loss) also is currently still unclear. Taken together, the findings provide hints that female joints may be anatomically (and mechanically) disadvantaged and at greater risk of incident knee OA.

Very few clinical studies mention the term “sex” in the title or abstract, whereas most often these are “hidden” in the result section, if at all present. When mixed cohorts are studied, sex is often treated as a confounder, but no descriptive or statistical analysis is undertaken in sex-specific strata. If sex-comparisons are made, they are often confounded by differences in age, BMI, RKOA stage, or other. Statistical adjustment is helpful, but matching is more effective. Only one paper reported sex-differences of a structure modifying intervention, but this unfortunately is not standard at all in clinical trials.

Further, there is an important lack of sex-specific healthy reference data. Given that most modern imaging modalities are non-invasive, initiatives should be taken to build larger reference data bases of articular tissue properties at various developmental

and maturity stages in healthy women and men. These findings should be made publicly available, as has been done for cohorts with risk factors or presence of OA.

Whereas some articular tissue measures are independent of body height, others are not. In these, “observed” and “genuine” sex-differences must be differentiated, the latter potentially revealed by studies matching women and men for identical height. Alternatively, normalization must be undertaken, but normalization is particularly challenging when comparing longitudinal change between both sexes. Depending on the scientific question or anatomical entity, it is better to capture change in absolute or relative (%) terms, with absolute change potentially underemphasizing change in women. Statistical adjustment for baseline values may render sex-specific analysis to be dominated by baseline sex-differences, assuming the character of a cross-sectional comparison. We therefore recommend to always express sex-differences in longitudinal change in both absolute and relative terms, and to always provide crude longitudinal observations before presenting adjusted values, and preferably, these different approaches should be pointing in the same direction, before solid conclusions are drawn.

Clearly, the “one size/sex fits all” approach must be abandoned in OA research and scientific reporting. Differences between women, men, and other biologically or socially categorized individuals must be recognized[98], both in research and in clinical practice. Any OA research study in mixed samples, preclinical or clinical, observational or interventional, should mention the existence of sex-specific results in the abstract, and report sex-stratified data at least in a supplement. Statistical analysis should be stratified between women and men, after performing sex-adjusted analyses

in the full sample, at least as a pre-specified secondary or post-hoc analysis. This approach should be pursued per self-commitment of the research community, and should gradually be adopted into official editorial policies of scientific journals and text-books for full implementation.

**Acknowledgement:** We thank Sophia Eckstein, Susanne Maschek and Anna Wisser for proofreading and editing the text. We would further like to thank the following colleagues, in alphabetic order, for their help in identifying or interpreting relevant literature for this review article, who have kindly agreed to be mentioned in context of their contribution to this review: **Francis Berenbaum**, Department of Rheumatology, Sorbonne University, INSERM, AP-HP Saint-Antoine Hospital, Paris, France**; Flavia Cicuttini**, School of Public Health and Preventive Medicine, Monash University, Melbourne, Australia**; Adam Culvenor**, La Trobe University School of Allied Health, Bundoora, Australia**; Martin Englund**, Clinical Epidemiology Unit, Orthopedics, Department of Clinical Sciences Lund, Lund University, Lund, Sweden**; Ali Guermazi**, Department of Radiology, Boston University School of Medicine, Boston, USA**; David Hunter**, Sydney Musculoskeletal Health, Kolling Institute, Faculty of Medicine and Health, The University of Sydney, and Rheumatology Department, Royal North Shore Hospital, St Leonards, Sydney, Australia**.; Mylene Jansen**, Rheumatology & Clinical Immunology, University Medical Center Utrecht, Utrecht, The Netherlands**; Gabby Joseph**, Musculoskeletal & Quantitative Imaging Research Group, Department of Radiology and Biomedical Imaging, University of California

San Francisco, CA, USA**; Thomas Link**, Musculoskeletal & Quantitative Imaging Research Group, Department of Radiology and Biomedical Imaging, University of California San Francisco, CA, USA**; Malcolm Logan**, Randall Centre for Cell and Molecular Biophysics King's College London, Guy's Campus, London, UK**; Michael Nevitt**, Epidemiology & Biostatistics, School of Medicine, University of California, San Francisco, CA, USA**; Frank Roemer**, Department of Radiology, Universitätsklinikum Erlangen & Friedrich-Alexander Universität Erlangen-Nürnberg (FAU), Erlangen, Germany**.**

**References:**


1. Hu W, Hao Z, Du P, Di Vincenzo F, Manzi G, Cui J, et al. Genomic inference of a severe human bottleneck during the Early to Middle Pleistocene transition. *Science*. 2023;381(6661):979-984.
2. Pietsch TW. Dimorphism, parasitism, and sex revisited: modes of reproduction among deep-sea ceratioid anglerfishes (Teleostei: Lophiiformes). *Ichthyol Res*. 2005;52(3):207-236.
3. McCormick SK, Holekamp KE, Smale L, Weldele ML, Glickman SE, Place NJ. Sex Differences in Spotted Hyenas. *Cold Spring Harb Perspect Biol*. 2022;14(6).
4. Overstreet DS, Strath LJ, Jordan M, Jordan IA, Hobson JM, Owens MA, et al. A Brief Overview: Sex Differences in Prevalent Chronic Musculoskeletal Conditions. *Int J Environ Res Public Health*. 2023;20(5).
5. Tschon M, Contartese D, Pagani S, Borsari V, Fini M. Gender and Sex Are Key

Determinants in Osteoarthritis Not Only Confounding Variables. A Systematic Review of Clinical Data. *J Clin Med*. 2021;10(14).

6. Laitner MH, Erickson LC, Ortman E. Understanding the Impact of Sex and Gender in Osteoarthritis: Assessing Research Gaps and Unmet Needs. *J Womens Health (Larchmt)*. 2021;30(5):634-641.

7. Hunter DJ, Bierma-Zeinstra S. Osteoarthritis. *Lancet*. 2019;393(10182):1745-1759.

8. Zhang Y, Xu L, Nevitt MC, Aliabadi P, Yu W, Qin M, et al. Comparison of the prevalence of knee osteoarthritis between the elderly Chinese population in Beijing and whites in the United States: The Beijing Osteoarthritis Study. *Arthritis Rheum*. 2001;44(9):2065-2071.

9. Felson DT, Naimark A, Anderson J, Kazis L, Castelli W, Meenan RF. The prevalence of knee osteoarthritis in the elderly. The Framingham Osteoarthritis Study. *Arthritis Rheum*. 1987;30(8):914-918.

10. Eaton CB, Schaefer LF, Duryea J, Driban JB, Lo GH, Roberts MB, et al. Prevalence, Incidence, and Progression of Radiographic and Symptomatic Hand Osteoarthritis: The Osteoarthritis Initiative. *Arthritis Rheumatol (Hoboken, NJ)*. 2022;74(6):992-1000.

11. Fan Z, Yan L, Liu H, Li X, Fan K, Liu Q, et al. The prevalence of hip osteoarthritis: a systematic review and meta-analysis. *Arthritis Res Ther*. 2023;25(1):51.

12. Hussain SM, Cicuttini FM, Alyousef B, Wang Y. Female hormonal factors and osteoarthritis of the knee, hip and hand: a narrative review. *Climacteric*.

2018;21(2):132-139.

13. Peshkova M, Lychagin A, Lipina M, Di Matteo B, Anzillotti G, Ronzoni F, et al. Gender-Related Aspects in Osteoarthritis Development and Progression: A Review. *Int J Mol Sci*. 2022;23(5).

14. Nguyen U-SDT, Saunders FR, Martin KR. Sex Difference in OA: Should We Blame Estrogen? *Eur J Rheumatol*. January 2023.

15. Black AL, Clark AL. Sexual dimorphism in knee osteoarthritis: Biomechanical variances and biological influences. *J Orthop*. 2022;32:104-108.

16. Szilagyi IA, Waarsing JH, van Meurs JBJ, Bierma-Zeinstra SMA, Schiphof D. A systematic review of the sex differences in risk factors for knee osteoarthritis. *Rheumatology (Oxford)*. 2023;62(6):2037-2047.

17. Maleki-Fischbach M, Jordan JM. New developments in osteoarthritis. Sex differences in magnetic resonance imaging-based biomarkers and in those of joint metabolism. *Arthritis Res Ther*. 2010;12(4):212.

18. Contartese D, Tschon M, De Mattei M, Fini M. Sex Specific Determinants in Osteoarthritis: A Systematic Review of Preclinical Studies. *Int J Mol Sci*. 2020;21(10).

19. Hwang HS, Park IY, Hong JI, Kim JR, Kim HA. Comparison of joint degeneration and pain in male and female mice in DMM model of osteoarthritis. *Osteoarthr Cartil*. 2021;29(5):728-738.

20. Ro JY, Zhang Y, Tricou C, Yang D, da Silva JT, Zhang R. Age and Sex Differences in Acute and Osteoarthritis-Like Pain Responses in Rats. *J Gerontol A Biol Sci Med Sci*. 2020;75(8):1465-1472.

21. Peterfy C, Li J, Zaim S, Duryea J, Lynch J, Miaux Y, et al. Comparison of fixed-flexion positioning with fluoroscopic semi-flexed positioning for quantifying radiographic joint-space width in the knee: test-retest reproducibility. *Skelet Radiol*. 2003;32(3):128-132.

22. Duryea J, Li J, Peterfy CG, Gordon C, Genant HK. Trainable rule-based algorithm for the measurement of joint space width in digital radiographic images of the knee. *Med Phys*. 2000;27(2000):580-591.

23. Eckstein F, Boudreau R, Wang Z, Hannon MJ, Duryea J, Wirth W, et al. Comparison of radiographic joint space width and magnetic resonance imaging for prediction of knee replacement: A longitudinal case-control study from the Osteoarthritis Initiative. *EurRadiol*. 2016;26(6):1942-1951.

24. FDA. Guidance for industry on clinical development programs for drugs, devices and biological products intended for the treatment of osteoarthritis (OA). www.fda.gov/.../GuidanceComlianceRegulatoryInformation/GUidances/ucm071577.pdf. Published 1999.

25. Eckstein F, Hochberg MC, Guehring H, Moreau F, Ona V, Bihlet AR, et al. Long-term structural and symptomatic effects of intra-articular sprifermin in patients with knee osteoarthritis: 5-year results from the FORWARD study. *Ann Rheum Dis*. 2021;80(8):1062-1069.

26. Beattie KA, Duryea J, Pui M, O’Neill J, Boulos P, Webber CE, et al. Minimum joint space width and tibial cartilage morphology in the knees of healthy individuals: a cross-sectional study. *BMC Musculoskelet Disord*. 2008;9:119.

27. Eckstein F, Wirth W, Nevitt MC. Recent advances in osteoarthritis imaging-the Osteoarthritis Initiative. *Nat Rev Rheumatol*. 2012;8(10):622-630.

28. Eckstein F, Kwoh CK, Link TM. Imaging research results from the Osteoarthritis Initiative (OAI): a review and lessons learned 10 years after start of enrolment. *Ann Rheum Dis*. 2014;73(7):1289-1300.

29. Altman RD, Gold GE. Atlas of individual radiographic features in osteoarthritis, revised. *Osteoarthr Cartil*. 2007;15(Suppl A):A1--56.

30. Roth M, Wirth W, Emmanuel K, Culvenor AG, Eckstein F. The contribution of 3D quantitative meniscal and cartilage measures to variation in normal radiographic joint space width—Data from the Osteoarthritis Initiative healthy reference cohort. *Eur J Radiol*. 2017;87:90-98.

31. Bloecker K, Englund M, Wirth W, Hudelmaier M, Burgkart R, Frobell RB, et al. Revision 1 size and position of the healthy meniscus, and its correlation with sex, height, weight, and bone area- a cross-sectional study. *BMC Musculoskelet Disord*. 2011;12:248.

32. Wenger A, Englund M, Wirth W, Hudelmaier M, Kwoh K, Eckstein F. Relationship of 3D meniscal morphology and position with knee pain in subjects with knee osteoarthritis: a pilot study. *Eur Radiol*. 2012;22(1):211-220.

33. Roth M, Emmanuel K, Wirth W, Kwoh CK, Hunter DJ, Eckstein F. Sensitivity to change and association of three-dimensional meniscal measures with radiographic joint space width loss in rapid clinical progression of knee osteoarthritis. *Eur Radiol*. 2018;28:1844-1853.

34. Englund M, Guermazi A, Gale D, Hunter DJ, Aliabadi P, Clancy M, et al.

Incidental meniscal findings on knee MRI in middle-aged and elderly persons. *NEnglJMed*. 2008;359(11):1108-1115.

35. Peterfy C, Guermazi A, Zaim S. Whole-organ magnetic resonance imaging score (WORMS) of the knee in osteoarthritis. *OsteoarthritisCartilage*. 2003;(submitted.

36. Guermazi A, Niu J, Hayashi D, Roemer FW, Englund M, Neogi T, et al. Prevalence of abnormalities in knees detected by MRI in adults without knee osteoarthritis: population based observational study (Framingham Osteoarthritis Study). *BMJ*. 2012;345:e5339.

37. Svensson F, Felson DT, Zhang F, Guermazi A, Roemer FW, Niu J, et al. Meniscal body extrusion and cartilage coverage in middle-aged and elderly without radiographic knee osteoarthritis. *Eur Radiol*. 2019;29(4):1848-1854.

38. Stehling C, Lane NE, Nevitt MC, Lynch J, McCulloch CE, Link TM. Subjects with higher physical activity levels have more severe focal knee lesions diagnosed with 3T MRI: analysis of a non-symptomatic cohort of the osteoarthritis initiative. *Osteoarthritis Cartilage*. 2010;18(6):776-786.

39. Kumar D, Souza RB, Subburaj K, MacLeod TD, Singh J, Calixto NE, et al. Are There Sex Differences in Knee Cartilage Composition and Walking Mechanics in Healthy and Osteoarthritis Populations? *ClinOrthopRelat Res*. 2015;473(8):2548-2558.

40. Hashemi J, Chandrashekar N, Mansouri H, Slauterbeck JR, Hardy DM. The human anterior cruciate ligament: sex differences in ultrastructure and correlation with biomechanical properties. *J Orthop Res*. 2008;26(7):945-950.

41. Onambélé GNL, Burgess K, Pearson SJ. Gender-specific in vivo measurement of the structural and mechanical properties of the human patellar tendon. *J Orthop Res*. 2007;25(12):1635-1642.

42. Shim JJ, Ateshian GA. A Hybrid Biphasic Mixture Formulation for Modeling Dynamics in Porous Deformable Biological Tissues. *Arch Appl Mech = Ingenieur-Archiv*. 2022;92(2):491-511.

43. Jones G, Glisson M, Hynes K, Cicuttini F. Sex and site differences in cartilage development: a possible explanation for variations in knee osteoarthritis in later life. *Arthritis Rheum*. 2000;43(0004-3591):2543-2549.

44. Jones G, Ding C, Glisson M, Hynes K, Ma D, Cicuttini F. Knee articular cartilage development in children: a longitudinal study of the effect of sex, growth, body composition, and physical activity. *PediatrRes*. 2003;54(0031-3998):230-236.

45. Spannow AH, Pfeiffer-Jensen M, Andersen NT, Herlin T, Stenbog E. Ultrasonographic measurements of joint cartilage thickness in healthy children: age- and sex-related standard reference values. *JRheumatol*. 2010;37(0315-162X (Print)):2595-2601.

46. Eckstein F, Boeth H, Diederichs G, Wirth W, Hudelmaier M, Cotofana S, et al. Longitudinal change in femorotibial cartilage thickness and subchondral bone plate area in male and female adolescent vs. mature athletes. *Ann Anat*. 2014;196(2-3):150-157.

47. Culvenor AG, Wirth W, Maschek S, Boeth H, Diederichs G, Duda G, et al. Longitudinal change in patellofemoral cartilage thickness, cartilage T2

relaxation times, and subchondral bone plate area in adolescent vs mature athletes. *Eur J Radiol*. 2017;92:24-29.

48. Eckstein F, Wirth W, Lohmander LS, Hudelmaier MI, Frobell RB. Five-year followup of knee joint cartilage thickness changes after acute rupture of the anterior cruciate ligament. *Arthritis Rheumatol*. 2015;67(1):152-161.

49. Culvenor AG, Eckstein F, Wirth W, Lohmander LS, Frobell R. Loss of patellofemoral cartilage thickness over 5 years following ACL injury depends on the initial treatment strategy: results from the KANON trial. *Br J Sports Med*. February 2019:bjsports--2018--100167.

50. Cicuttini F, Forbes A, Morris K, Darling S, Bailey M, Stuckey S. Gender differences in knee cartilage volume as measured by magnetic resonance imaging. *OsteoarthritisCartilage*. 1999;7(1063-4584):265-271.

51. Ding C, Cicuttini F, Scott F, Glisson M, Jones G. Sex differences in knee cartilage volume in adults: role of body and bone size, age and physical activity. *Rheumatol*. 2003;(1462-0324).

52. Antony B, Venn A, Cicuttini F, March L, Blizzard L, Dwyer T, et al. Association of Body Composition and Hormonal and Inflammatory Factors With Tibial Cartilage Volume and Sex Difference in Cartilage Volume in Young Adults. *Arthritis Care Res (Hoboken)*. 2016;68(4):517-525.

53. Berry PA, Wluka AE, Davies-Tuck ML, Wang Y, Strauss BJ, Dixon JB, et al. Sex differences in the relationship between bone mineral density and tibial cartilage volume. *Rheumatology (Oxford)*. 2011;50(3):563-568.

54. Cicuttini FM, Wluka A, Bailey M, O’Sullivan R, Poon C, Yeung S, et al.

Factors affecting knee cartilage volume in healthy men. *Rheumatology (Oxford)*. 2003;42(2):258-262.

55. Hanna F, Ebeling PR, Wang Y, O'Sullivan R, Davis S, Wluka AE, et al. Factors influencing longitudinal change in knee cartilage volume measured from magnetic resonance imaging in healthy men. *AnnRheumDis*. 2005;64(7):1038-1042.

56. Faber SC, Eckstein F, Lukasz S, Mühlbauer R, Hohe J, Englmeier KH, et al. Gender differences in knee joint cartilage thickness, volume and articular surface areas: assessment with quantitative three-dimensional MR imaging. *Skelet Radiol*. 2001;30(0364-2348):144-150.

57. Eckstein F, Siedek V, Glaser C, Al-Ali D, Englmeier K-HH, Reiser M, et al. Correlation and sex differences between ankle and knee cartilage morphology determined by quantitative magnetic resonance imaging. *Ann Rheum Dis*. 2004;63(11):1490-1495.

58. Otterness IG, Eckstein F. Women have thinner cartilage and smaller joint surfaces than men after adjustment for body height and weight. *Osteoarthr Cart*. 2007;15(6):666-672.

59. Otterness IG, Le Graverand M-PHP, Eckstein F. Allometric relationships between knee cartilage volume, thickness, surface area and body dimensions. *Osteoarthr Cart*. 2008;16(1):34-40.

60. Eckstein F, Yang M, Guermazi A, Roemer FW, Hudelmaier M, Picha K, et al. Reference values and Z-scores for subregional femorotibial cartilage thickness--results from a large population-based sample (Framingham) and comparison

with the non-exposed Osteoarthritis Initiative reference cohort. *Osteoarthr Cartil*. 2010;18(10):1275-1283.

61. Frobell RB, Nevitt MC, Hudelmaier M, Wirth W, Wyman BT, Benichou O, et al. Femorotibial subchondral bone area and regional cartilage thickness: a cross-sectional description in healthy reference cases and various radiographic stages of osteoarthritis in 1,003 knees from the Osteoarthritis Initiative. *Arthritis Care Res(Hoboken)*. 2010;62(11):1612-1623.

62. Eckstein F, Faber S, Muhlbauer R, Hohe J, Englmeier KH, Reiser M, et al. Functional adaptation of human joints to mechanical stimuli. *OsteoarthritisCartilage*. 2002;10(1063-4584):44-50.

63. Bricca A, Wirth W, Juhl CB, Kemnitz J, Hunter DJ, Kwoh CK, et al. Moderate Physical Activity and Prevention of Cartilage Loss in People With Knee Osteoarthritis: Data From the Osteoarthritis Initiative. *Arthritis Care Res (Hoboken)*. 2019;71(2):218-226.

64. Roemer FW, Felson DT, Stefanik JJ, Rabasa G, Wang N, Crema MD, et al. Heterogeneity of cartilage damage in Kellgren and Lawrence grade 2 and 3 knees: the MOST study. *Osteoarthr Cartil*. 2022;30(5):714-723.

65. Frobell RB, Wirth W, Nevitt M, Wyman BT, Benichou O, Dreher D, et al. Presence, location, type and size of denuded areas of subchondral bone in the knee as a function of radiographic stage of OA - data from the OA initiative. *OsteoarthritisCartilage*. 2010;18(5):668-676.

66. Eckstein F, Wirth W, Hudelmaier M, Maschek S, Hitzl W, Wyman BT, et al. Relationship of compartment-specific structural knee status at baseline with

change in cartilage morphology: a prospective observational study using data from the osteoarthritis initiative. *Arthritis Res Ther*. 2009;11(3):R90.

67. Cotofana S, Wyman BT, Benichou O, Dreher D, Nevitt M, Gardiner J, et al. Relationship between knee pain and the presence, location, size and phenotype of femorotibial denuded areas of subchondral bone as visualized by MRI. *OsteoarthritisCartilage*. 2013;21(1522-9653 (Electronic)):1214-1222.

68. Buck RJ, Wirth W, Dreher D, Nevitt M, Eckstein F. Frequency and spatial distribution of cartilage thickness change in knee osteoarthritis and its relation to clinical and radiographic covariates - data from the osteoarthritis initiative. *Osteoarthr Cartil*. 2013;21(1):102-109.

69. Ding C, Cicuttini F, Blizzard L, Scott F, Jones G. A longitudinal study of the effect of sex and age on rate of change in knee cartilage volume in adults. *Rheumatology (Oxford)*. 2007;46(2):273-279.

70. Hanna FS, Teichtahl AJ, Wluka AE, Wang Y, Urquhart DM, English DR, et al. Women have increased rates of cartilage loss and progression of cartilage defects at the knee than men: a gender study of adults without clinical knee osteoarthritis. *Menopause*. 2009;16(4):666-670.

71. Ding C, Martel-Pelletier J, Pelletier JP, Abram F, Raynauld JP, Cicuttini F, et al. Two-year prospective longitudinal study exploring the factors associated with change in femoral cartilage volume in a cohort largely without knee radiographic osteoarthritis. *OsteoarthritisCartilage*. 2008;16(1063-4584 (Print)):443-449.

72. Cicuttini F, Wluka A, Wang Y, Stuckey S. The determinants of change in

patella cartilage volume in osteoarthritic knees. *J Rheumatol*. 2002;29(0315-162X):2615-2619.

73. Teichtahl AJ, Wang Y, Wluka AE, Szramka M, English DR, Giles GG, et al. The longitudinal relationship between body composition and patella cartilage in healthy adults. *Obesity (Silver Spring)*. 2008;16(2):421-427.

74. Cai G, Jiang M, Cicuttini F, Jones G. Association of age, sex and BMI with the rate of change in tibial cartilage volume: a 10.7-year longitudinal cohort study. *Arthritis Res Ther*. 2019;21(1):273.

75. de Klerk BM, Schiphof D, Groeneveld FPMJ, Koes BW, van Osch GJVM, van Meurs JBJ, et al. No clear association between female hormonal aspects and osteoarthritis of the hand, hip and knee: a systematic review. *Rheumatology (Oxford)*. 2009;48(9):1160-1165.

76. Hanna FS, Bell RJ, Cicuttini FM, Davison SL, Wluka AE, Davis SR. The relationship between endogenous testosterone, preandrogens, and sex hormone binding globulin and knee joint structure in women at midlife. *Semin Arthritis Rheum*. 2007;37(1):56-62.

77. Jin X, Wang BH, Wang X, Antony B, Zhu Z, Han W, et al. Associations between endogenous sex hormones and MRI structural changes in patients with symptomatic knee osteoarthritis. *Osteoarthr Cartil*. 2017;25(7):1100-1106.

78. Wluka AE, Davis SR, Bailey M, Stuckey SL, Cicuttini FM. Users of oestrogen replacement therapy have more knee cartilage than non-users. *AnnRheumDis*. 2001;60(0003-4967):332-336.

79. Cicuttini FM, Wluka AE, Wang Y, Stuckey SL, Davis SR. Effect of estrogen

replacement therapy on patella cartilage in healthy women. *ClinExpRheumatol*. 2003;21(1):79-82.

80. Wei S, Venn A, Ding C, Martel-Pelletier J, Pelletier J-P, Abram F, et al. The associations between parity, other reproductive factors and cartilage in women aged 50-80 years. *Osteoarthr Cartil*. 2011;19(11):1307-1313.

81. Wluka AE, Wolfe R, Davis SR, Stuckey S, Cicuttini FM. Tibial cartilage volume change in healthy postmenopausal women: a longitudinal study. *AnnRheum Dis*. 2004;63(0003-4967):444-449.

82. Tanamas SK, Wijethilake P, Wluka AE, Davies-Tuck ML, Urquhart DM, Wang Y, et al. Sex hormones and structural changes in osteoarthritis: a systematic review. *Maturitas*. 2011;69(2):141-156.

83. Link TM, Joseph GB, Li X. MRI-based T1rho and T2 cartilage compositional imaging in osteoarthritis: what have we learned and what is needed to apply it clinically and in a trial setting? *Skeletal Radiol*. 2023;52(11):2137-2147.

84. Mosher TJ, Dardzinski BJ. Cartilage MRI T2 relaxation time mapping: overview and applications. *SeminMusculoskeletRadiol*. 2004;8(4):355-368.

85. Mosher TJ, Collins CM, Smith HE, Moser LE, Sivarajah RT, Dardzinski BJ, et al. Effect of gender on in vivo cartilage magnetic resonance imaging T2 mapping. *JMagn Reson*. 2004;19(3):323-328.

86. Kim HK, Shiraj S, Anton CG, Horn PS, Dardzinski BJ. Age and sex dependency of cartilage T2 relaxation time mapping in MRI of children and adolescents. *AJR AmJ Roentgenol*. 2014;202(3):626-632.

87. Wirth W, Eckstein F, Boeth H, Diederichs G, Hudelmaier M, Duda GNN.

Longitudinal analysis of MR spin–spin relaxation times (T2) in medial femorotibial cartilage of adolescent vs mature athletes: dependence of deep and superficial zone properties on sex and age. *Osteoarthr Cartil*. 2014;22(10):1554-1558.

88. Joseph GB, McCulloch CE, Nevitt MC, Heilmeier U, Nardo L, Lynch JA, et al. A reference database of cartilage 3T MRI T2 values in knees without diagnostic evidence of cartilage degeneration: Data from the osteoarthritis initiative. *Osteoarthr Cartil*. 2015;23(6):897-905.

89. Wirth W, Maschek S, Eckstein F. Sex- and age-dependence of region- and layer-specific knee cartilage composition (spin–spin–relaxation time) in healthy reference subjects. *Ann Anat - Anat Anzeiger*. 2017;210(March):1-8.

90. Hudelmaier M, Glaser C, Hohe J, Englmeier KH, Reiser M, Putz R, et al. Age-related changes in the morphology and deformational behavior of knee joint cartilage. *Arthritis Rheum*. 2001;44(0004-3591):2556-2561.

91. Boocock M, McNair P, Cicuttini F, Stuart A, Sinclair T. The short-term effects of running on the deformation of knee articular cartilage and its relationship to biomechanical loads at the knee. *Osteoarthr Cartil*. 2009;17(7):883-890.

92. Gordon NC, Gear RW, Heller PH, Paul S, Miaskowski C, Levine JD. Enhancement of morphine analgesia by the GABAB agonist baclofen. *Neuroscience*. 1995;69(2):345-349.

93. Robinson ME, Riley JL, Brown FF, Gremillion H. Sex differences in response to cutaneous anesthesia: a double blind randomized study. *Pain*. 1998;77(2):143-149.

94. Gear RW, Gordon NC, Miaskowski C, Paul SM, Heller PH, Levine JD. Sexual dimorphism in very low dose nalbuphine postoperative analgesia. *Neurosci Lett*. 2003;339(1):1-4.

95. Hochberg MC, Guermazi A, Guehring H, Aydemir A, Wax S, Fleuranceau-Morel P, et al. Effect of Intra-Articular Sprifermin vs Placebo on Femorotibial Joint Cartilage Thickness in Patients With Osteoarthritis. *JAMA*. 2019;322(14):1360-1370.

96. van der Woude JAD, Welsing PM, van Roermund PM, Custers RJH, Kuchuk NO, Lafeber FPJGG. Prediction of cartilaginous tissue repair after knee joint distraction. *Knee*. 2016;23(5):792-795.

97. Jansen MP, van der Weiden GS, Van Roermund PM, Custers RJH, Mastbergen SC, Lafeber FPJG. Initial tissue repair predicts long-term clinical success of knee joint distraction as treatment for knee osteoarthritis. *Osteoarthr Cartil*. 2018;26(12):1604-1608.

98. Patel J, Chen S, Katzmeyer T, Pei YA, Pei M. Sex-dependent variation in cartilage adaptation: from degeneration to regeneration. *Biol Sex Differ*. 2023;14(1):17.

**Fig. 1**: Sagittal sections through the knee joint:

a) Medial femorotibial compartment;

b) Lateral femporotibial compartment;

c) Center of the knee joint;

d) 3 D reconstruction of articular knee tissues from MRI:

bones = beige; articular surfaces = white; meniscus = green;

ACL = blue; IPFP = bright yellow; muscle = red

**Fig. 2**: Posterior-anterior, fixed flexion, weight-bearing bilateral radiographs, with only the right knee shown;

a) medial JSN 0;

b) JSN 1;

c) JSN2;

d) JSN 3

**Fig. 3**: Bar graphs showing the mean (±SD) of minimum medial radiographic joint space width (mmJSW) in female and male OAI participants from the healthy reference cohort and participants with medial OARSI joint space narrowing (mJSN) grade 0, 1, 2, and 3.

**Fig. 4**: Menisci and ligaments:

a) Anatomical drawing showing the tibial plateau area and the anterior cruciate ligament (ACL) with origin and insertion;

b) 3D reconstruction of an MRI with several anatomical structures labelled:

menisci = red; articular cartilage = yellow; ACL = blue; PCL = green.

**Fig. 5** Subregional cartilage thickness reference values in men (above) and women (below) in the healty reference cohort of the OAI:

Subregions of the femorotibial joint:

Top: Weight-bearing portion of the femur seen from below

Middle: Medial (MFTC) and lateral (LFTC) femorotibial joint from posterior

Bottom: Tibial plateau seen from above

green = external; red= central; dárk blue = internal; tuquoise = anterior; yellow = posteerior

cLF = weight-bearing (central) lateral femur; LT = lateral tibia;

cMF = weight-bearing (central) medial femur; MT = medial tibia

*Adapted from Eckstein F, Yang M, Guermazi A, Roemer FW, Hudelmaier M, Picha K, et al. Reference values and Z-scores for subregional femorotibial cartilage thickness--results from a large population-based sample (Framingham) and comparison with the non-exposed Osteoarthritis Initiative reference cohort. Osteoarthr Cartil. 2010;18(10):1275-1283.*

**Fig. 6a** Subregional cartilage T2 reference values **(superficial** layer)

in men (above) and women (below) in the OAI healty reference cohort :

Subregions of the femorotibial joint:

Top: Weight-bearing portion of the femur seen from below

Middle: Medial (MFTC) and lateral (LFTC) femorotibial joint from posterior

Bottom: Tibial plateau seen from above

green = external; red= central; dárk blue = internal; tuquoise = anterior; yellow = posteerior

cLF = weight-bearing (central) lateral femur; LT = lateral tibia;

cMF = weight-bearing (central) medial femur; MT = medial tibia

*Adapted from Wirth W, Maschek S, Eckstein F. Sex- and age-dependence of region- and layer-specific*

*knee cartilage composition (spin–spin–relaxation time) in healthy reference subjects. Ann Anat - Anat Anzeiger. 2017;210(March):1-8.*

**Fig. 6b** Subregional cartilage T2 reference values (**deep** layer)

in men (above) and women (below) in the OAI healty reference cohort :

Subregions of the femorotibial joint:

Top: Weight-bearing portion of the femur seen from below

Middle: Medial (MFTC) and lateral (LFTC) femorotibial joint from posterior

Bottom: Tibial plateau seen from above

green = external; red= central; dárk blue = internal; tuquoise = anterior; yellow = posteerior

cLF = weight-bearing (central) lateral femur; LT = lateral tibia;

cMF = weight-bearing (central) medial femur; MT = medial tibia

*Adapted from Wirth W, Maschek S, Eckstein F. Sex- and age-dependence of region- and layer-specific knee cartilage composition (spin–spin–relaxation time) in healthy reference subjects. Ann Anat - Anat Anzeiger. 2017;210(March):1-8.*

**Fig. 7:** Patellar cartilage deformation in younger and older men and women. Older subjects displayed less deformation than younger ones, but no sex-differences were observed. *Adapted from Hudelmaier M, Glaser C, Hohe J, Englmeier KH, Reiser M, Putz R, et al. Age-related changes in the morphology and deformational behavior of knee joint cartilage. Arthritis Rheum. 2001;44(0004-3591):2556-2561*

 Click here to access/download;Figure;Figure1.tif

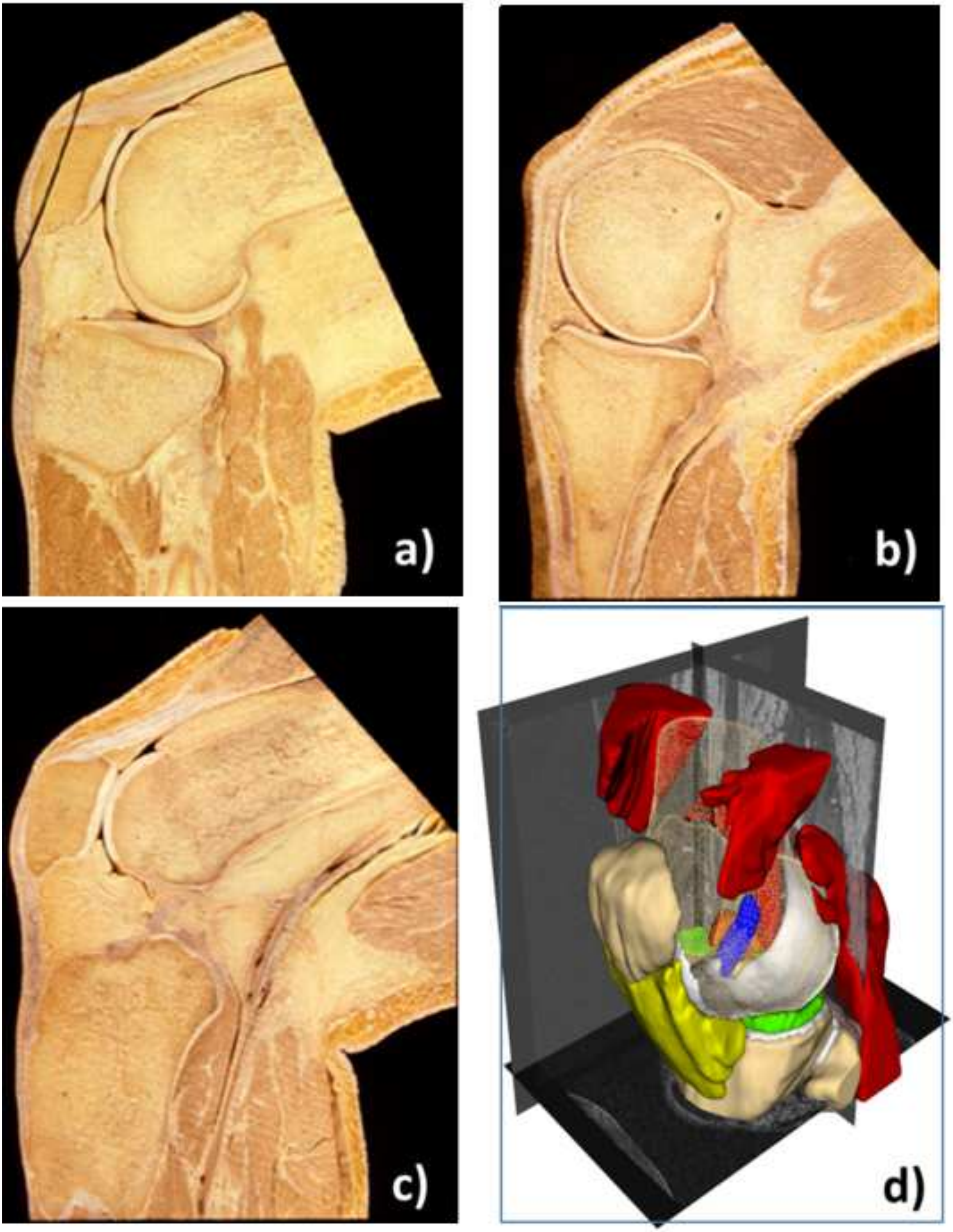

Figure 2

Click here to access/download;Figure;Figure2.tif

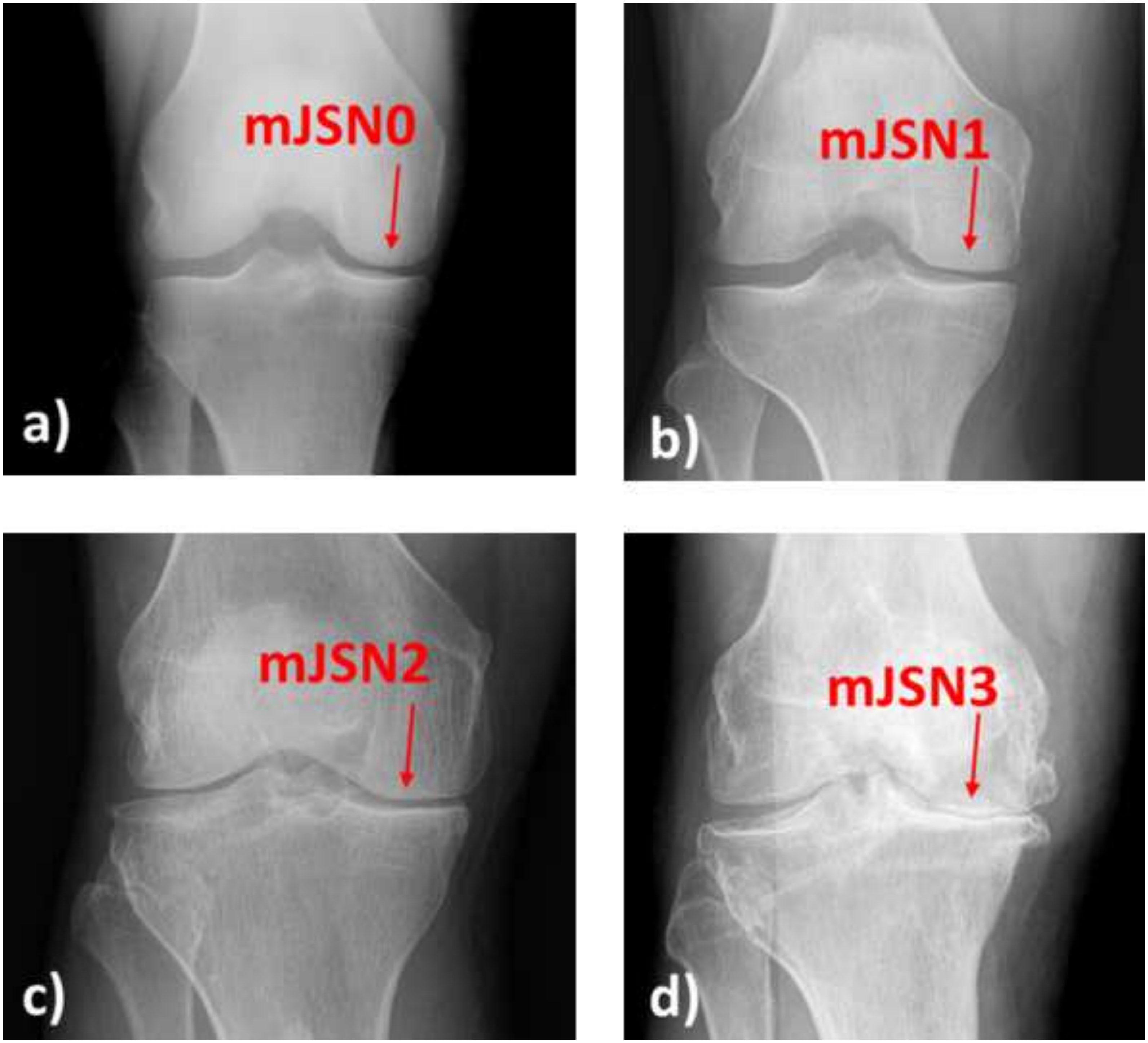

Figure 3

Click here to access/download;Figure;Figure3.tif

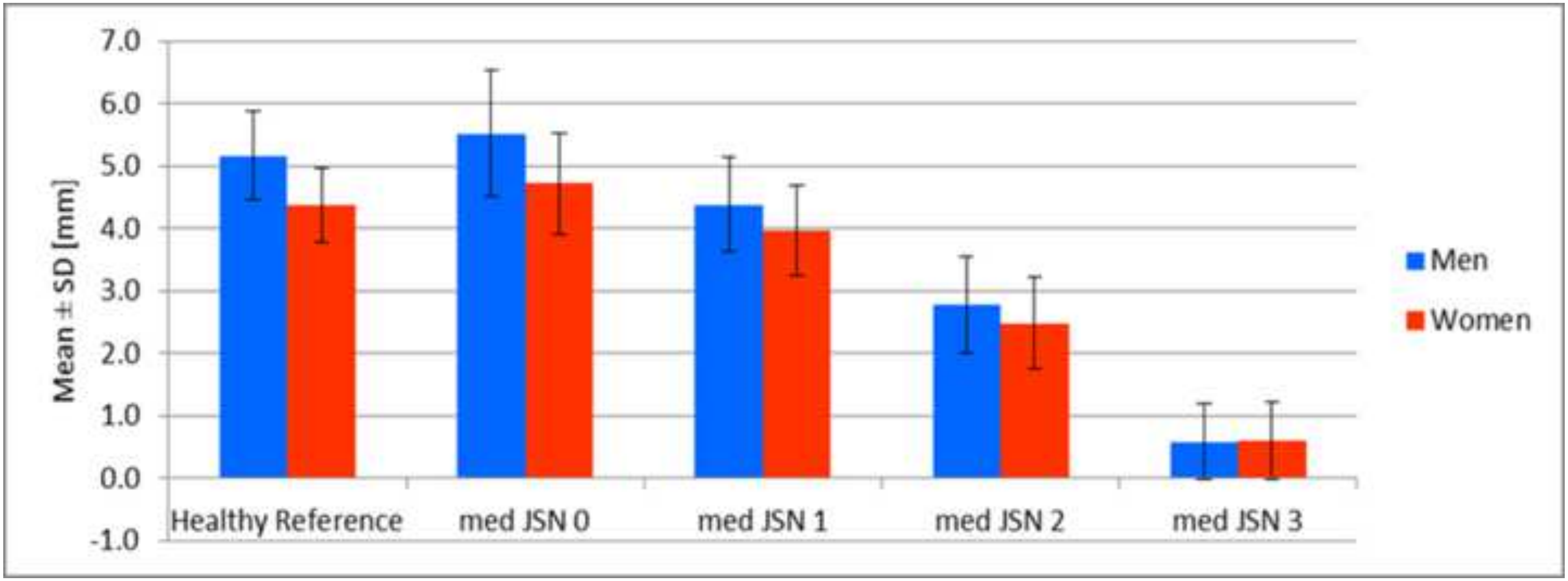

Figure 4

Click here to access/download;Figure;Figure4.tif

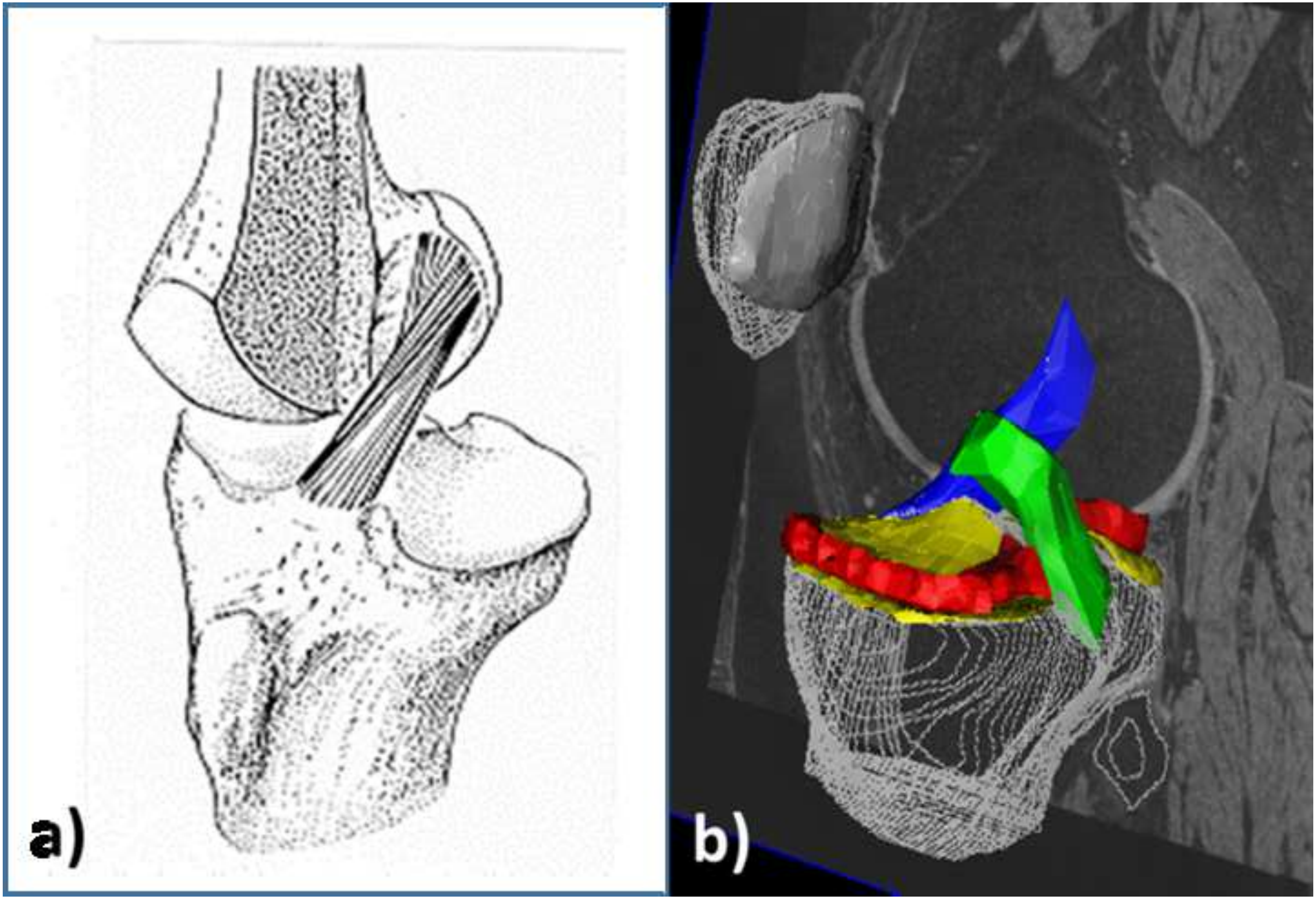

Figure 5

Click here to access/download;Figure;Figure5.tif

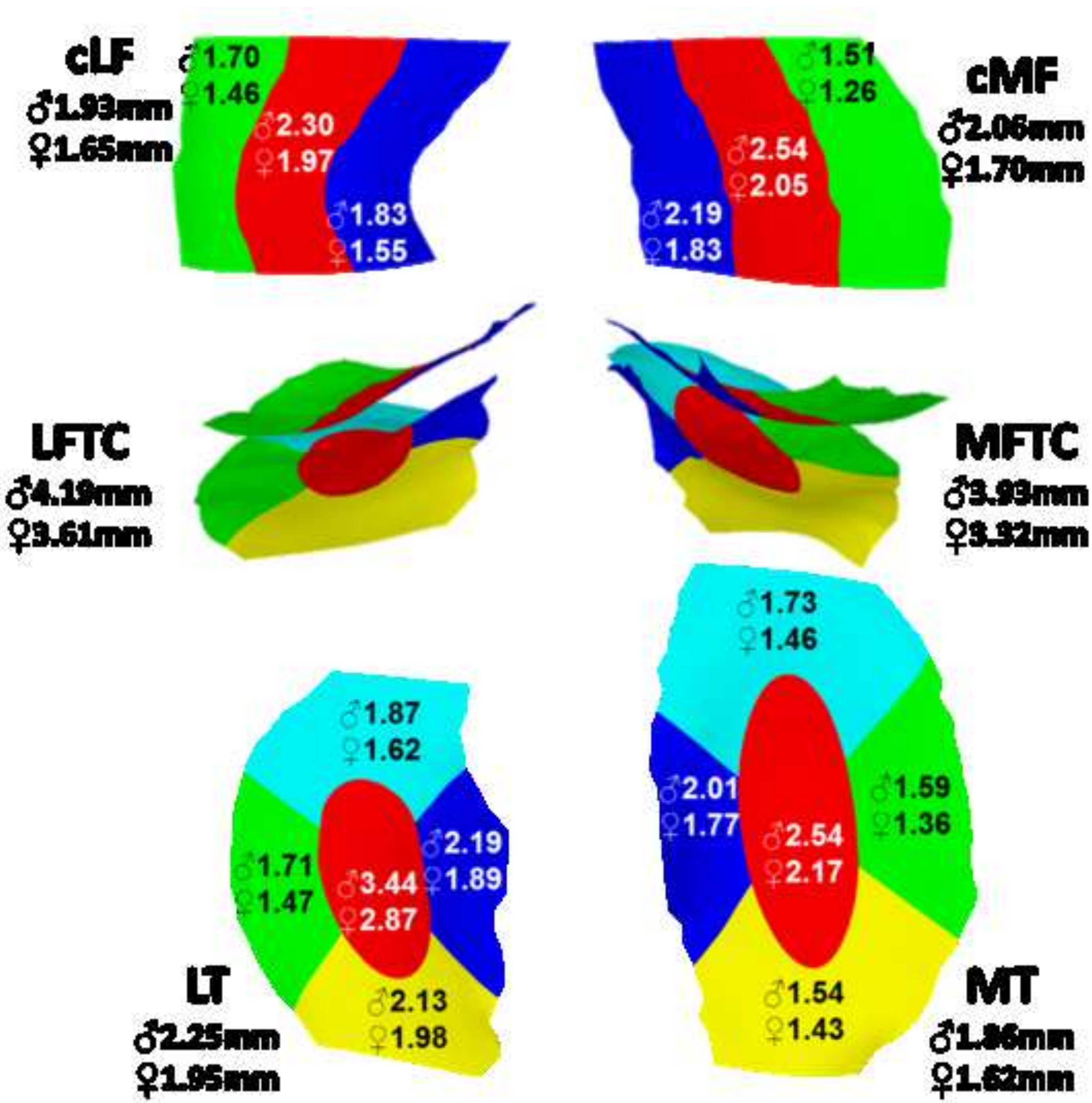

Figure 6a

Click here to access/download;Figure;Figure6a.tif

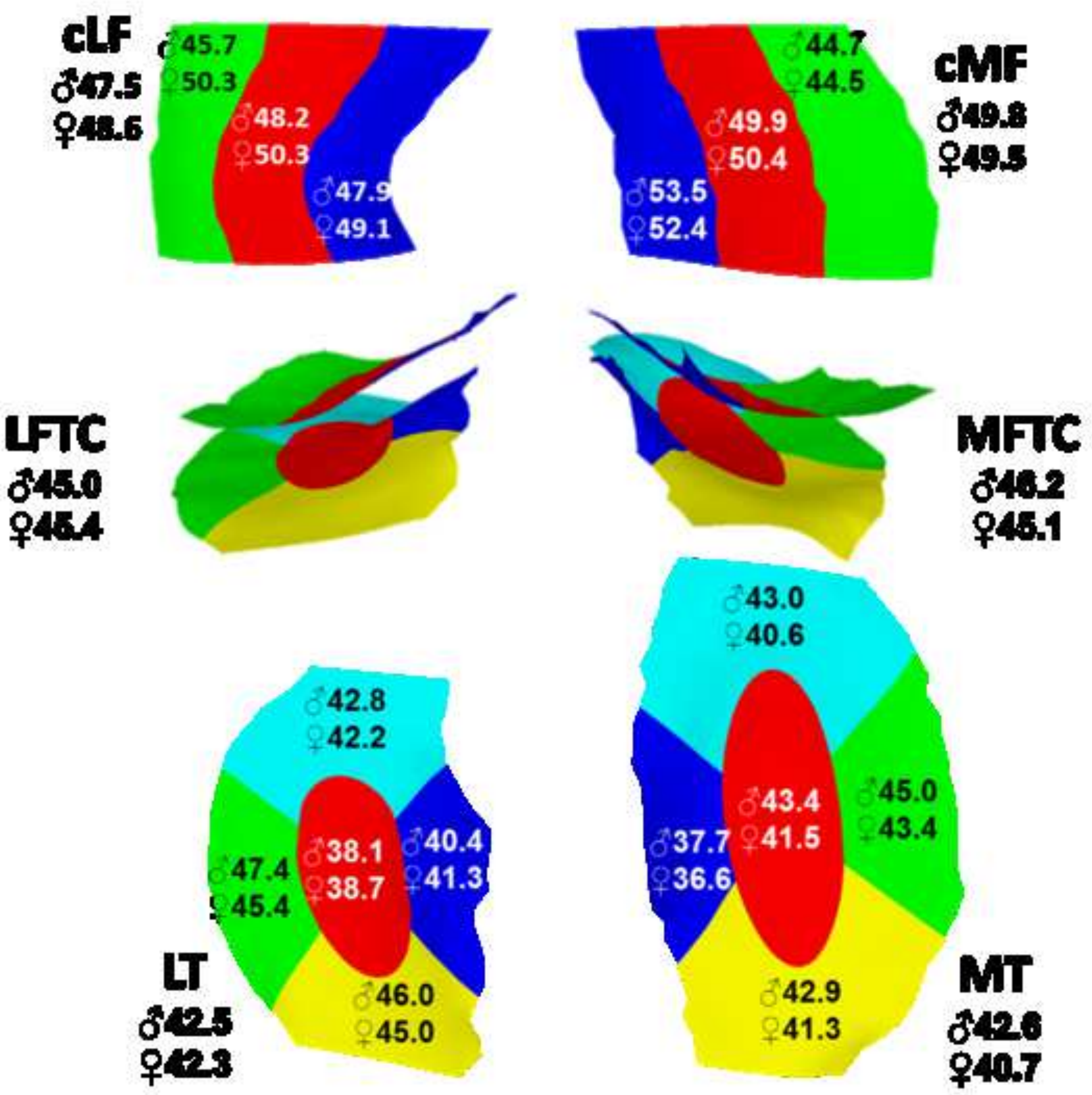

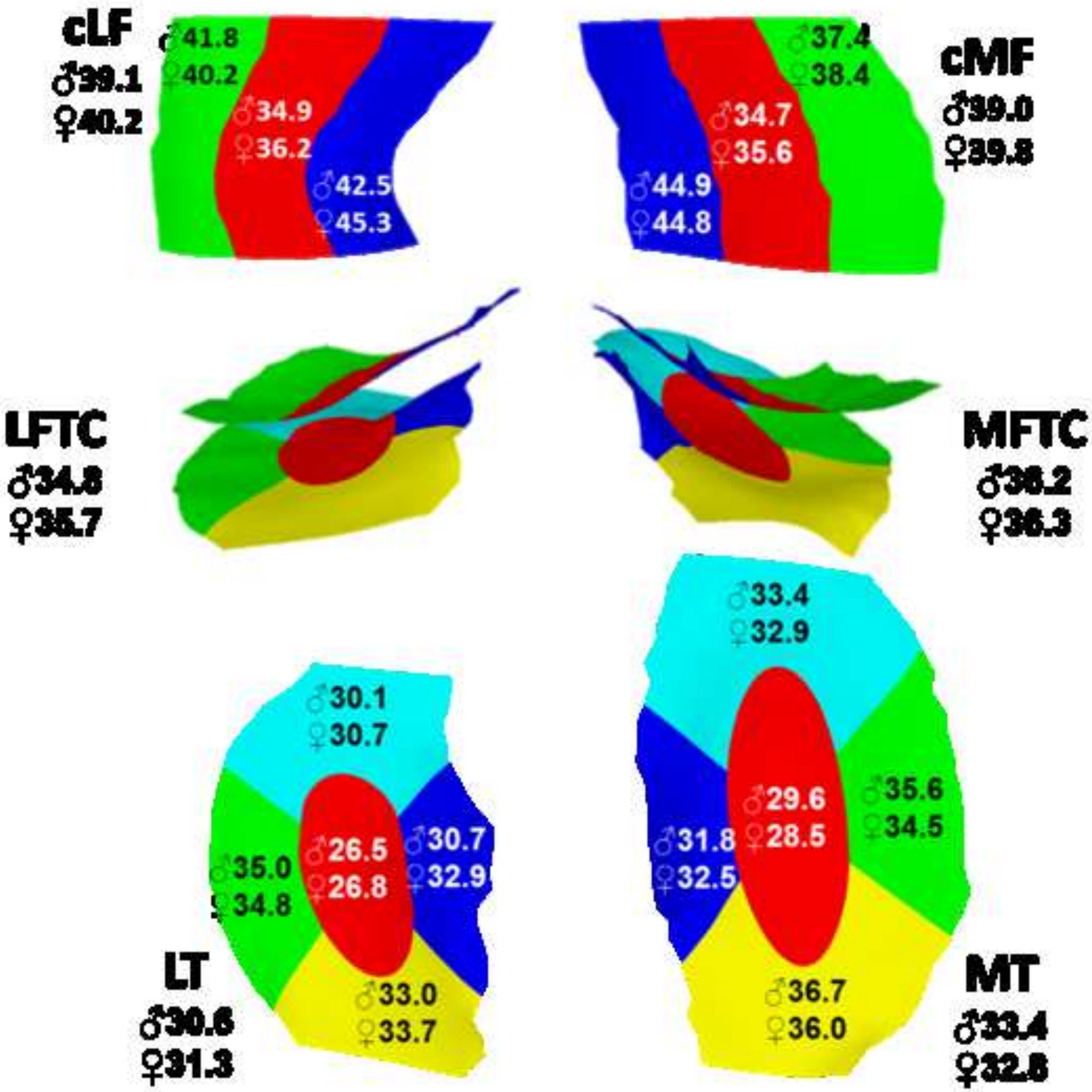
cLF
♂39.1
♀40.2
♂41.8
♀40.2
♂34.9
♀36.2
♂42.5
♀45.3
cMF
♂39.0
♀39.8
♂37.4
♀38.4
♂34.7
♀35.6
♂44.9
♀44.8
LFTC
♂34.8
♀35.7
MFTC
♂36.2
♀36.3
♂33.4
♀32.9
♂30.1
♀30.7
♂26.5
♀26.8
♂30.7
♀32.9
♂35.0
♀34.8
♂33.0
♀33.7
LT
♂30.6
♀31.3
♂29.6
♀28.5
♂31.8
♀32.5
♂35.6
♀34.5
♂36.7
♀36.0
MT
♂33.4
♀32.8

Figure 7

Click here to access/download;Figure;Figure7.tif

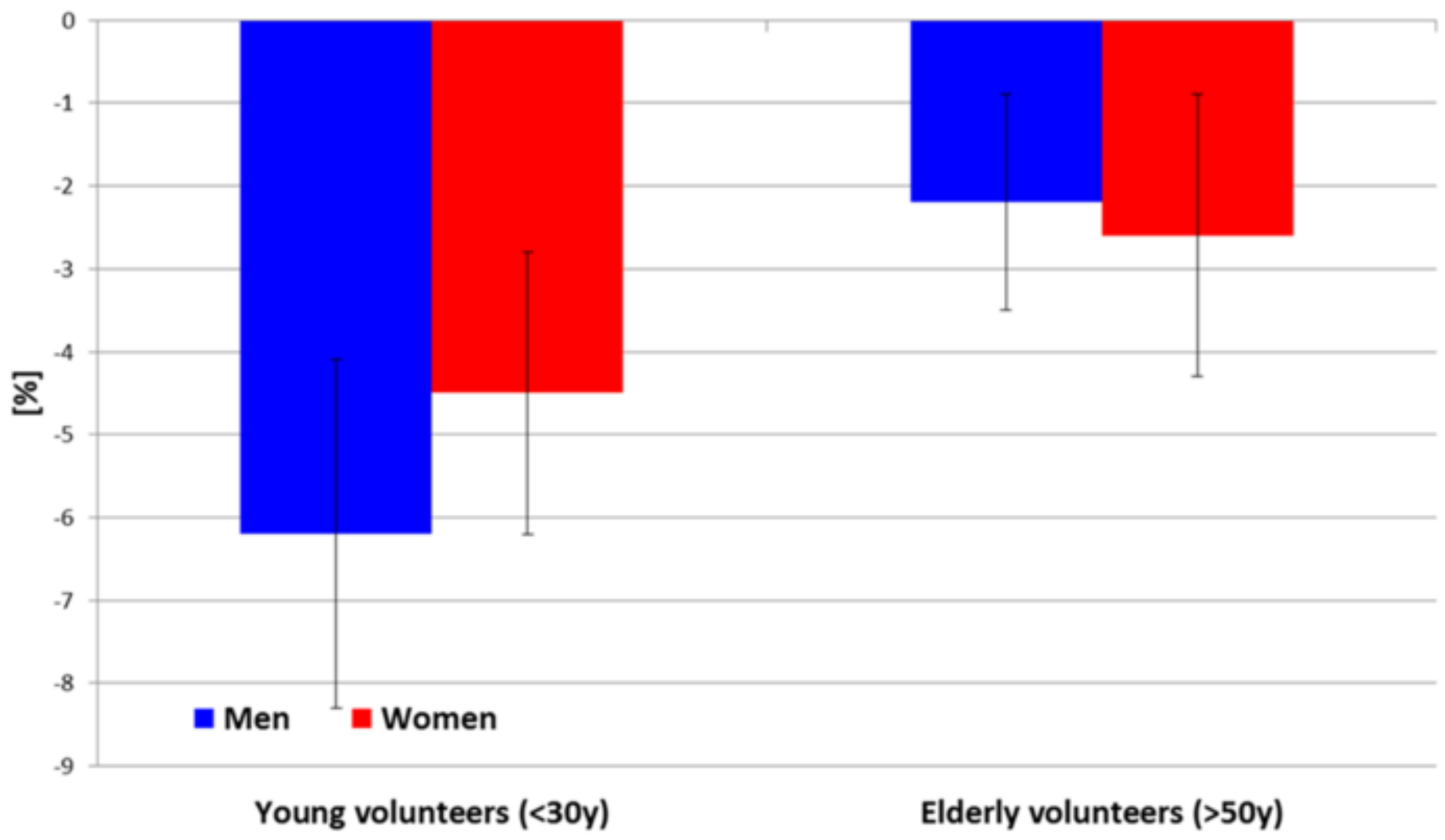

# *OSTEOARTHRITIS AND CARTILAGE*

# AUTHORS' DISCLOSURE

Manuscript title
**Sexual Dimorphism in Articular Tissue Anatomy – Key to Understanding Sex-differences in Osteoarthritis?**
Corresponding author: Felix Eckstein

**Authorship**
All authors should have made substantial contributions to all of the following: (1) the conception and design of the study, or acquisition of data, or analysis and interpretation of data, (2) drafting the article or revising it critically for important intellectual content, (3) final approval of the version to be submitted. By signing below each author also verifies that he (she) confirms that neither this manuscript, nor one with substantially similar content, has been submitted, accepted or published elsewhere (except as an abstract). Each manuscript must be accompanied by a declaration of contributions relating to sections (1), (2) and (3) above. This declaration should also name one or more authors who take responsibility for the integrity of the work as a whole, from inception to finished article. These declarations will be included in the published manuscript.

**Acknowledgement of other contributors**
All contributors who do not meet the criteria for authorship as defined above should be listed in an acknowledgements section. Examples of those who might be acknowledged include a person who provided purely technical help, writing assistance, or a department chair who provided only general support. Such contributors must give their consent to being named. Authors should disclose whether they had any writing assistance and identify the entity that paid for this assistance.

**Conflict of interest**
At the end of the text, under a subheading "Conflict of interest statement" all authors must disclose any financial and personal relationships with other people or organisations that could inappropriately influence (bias) their work. Examples of potential conflicts of interest include employment, consultancies, stock ownership, honoraria, paid expert testimony, patent applications/registrations, and research grants or other funding.

**Declaration of Funding**
All sources of funding should be declared as an acknowledgement at the end of the text.

**Role of the funding source**
Authors should declare the role of study sponsors, if any, in the study design, in the collection, analysis and interpretation of data; in the writing of the manuscript; and in the decision to submit the manuscript for publication. If the study sponsors had no such involvement, the authors should state this.

**Studies involving humans or animals**
Clinical trials or other experimentation on humans must be in accordance with the ethical standards of the responsible committee on human experimentation (institutional and national) *and* with the Helsinki Declaration of 1975, as revised in 2000. Randomized controlled trials should follow the Consolidated Standards of

Reporting Trials (CONSORT) guidelines and be registered in a public trials registry.

Studies involving experiments with animals were in accordance with institution guidelines

Please sign below to certify your manuscript complies with the above requirements and then upload this form at https://www.editorialmanager.com/oac/

| Author | Signature | Date |
|---|---|---|
| Felix Eckstein | 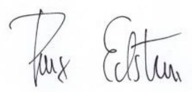 | 11/15/2023 |
| Reinhard Putz | 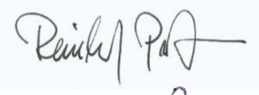 | 11/15/2023 |
| Wolfgang Wirth | 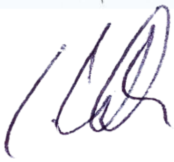 | 11/15/2023 |

Click here to access/download
**ICMJE COI form**
OAC_ICJME_disclosure F.E..docx

Click here to access/download
**ICMJE COI form**
OAC_ICJME_disclosure R.P..docx

Click here to access/download
**ICMJE COI form**
OAC_ICJME_disclosureWW.docx
